# Diagnosis and Treatment of Nontraumatic Osteonecrosis of the Femoral Head: A Systematic Review and Meta-Analyses for the ARCO Clinical Practice Guideline Development Workgroup

**DOI:** 10.3390/medsci14010107

**Published:** 2026-02-23

**Authors:** Romil R. Parikh, Alireza Mirzaei, Mary E. Butler, Diego J. Restrepo, Sergio F. Guarin Perez, Sallee Brandt, Gabrielle Swartz, Reza Katanbaf, Stuart B. Goodman, Michael A. Mont, Quanjun Cui, Lynne C. Jones, Edward Y. Cheng

**Affiliations:** 1 Minnesota Evidence-based Practice Center, Division of Health Policy & Management, University of Minnesota, Minneapolis, MN 55455, USA; 2 Department of Orthopaedic Surgery, University of Minnesota, Minneapolis, MN 55455, USA; 3 Department of Orthopaedic Surgery, Mayo Clinic, Rochester, MN 55905, USA; 4 Department of Orthopaedic Surgery, Sinai Hospital, LifeBridge Health, Baltimore, MD 21215, USA; 5 Department of Orthopaedic Surgery, Stanford University, Palo Alto, CA 94063, USA; 6 Department of Orthopaedic Surgery, University of Virginia, Charlottesville, VA 22903, USA; 7 Department of Orthopaedic Surgery, Johns Hopkins University, MD 21224, USA

**Keywords:** osteonecrosis, avascular necrosis, stem cells, core decompression, bone graft, osteotomy

## Abstract

Background/Objectives: Diagnostic evaluation and management of nontraumatic osteonecrosis of the femoral head (ONFH) vary substantially. This systematic review was conducted to inform development of the Association Research Circulation Osseous (ARCO) clinical practice guideline for diagnosis and treatment of ARCO stages I to III ONFH. Methods: We searched MEDLINE, EMBASE, Web of Science, SCOPUS, Global Index Medicus, and the Cochrane Library for studies evaluating imaging modalities and treatments for adult ONFH. We assessed risk of bias using the QUADAS-2, the ROB-2, and the ROBINS-I tools; conducted meta-analyses using random-effects regression; and evaluated certainty of evidence using GRADE methodology. Results: Among 36 included studies, 18 addressed diagnostic test accuracy and 18 addressed comparative effectiveness of treatments. Magnetic resonance imaging (MRI) demonstrated the highest pooled sensitivity (0.91; 95% confidence interval (CI), 0.87 to 0.94) and specificity (0.96; 95% CI, 0.87 to 0.99) for ONFH diagnosis. Bone marrow edema and grade 2+ joint effusion on MRI differentiated symptomatic versus asymptomatic disease. Computed tomography and MRI better detected subchondral fractures than plain radiography. Very low-grade evidence suggested lower rates of femoral head collapse with core decompression plus bone marrow concentrate compared with core decompression alone (pooled relative risk [RR], 0.55; 95% CI, 0.36 to 0.83), and with vascularized versus non-vascularized bone grafting (RR, 0.35; 95% CI, 0.14 to 0.84) over a ≤5-year follow-up. Based on three non-comparative case series, osteotomies might have a lower risk of collapse over a 10- to 20-year follow-up, but this needs to be evaluated in future comparative research. Inconsistent outcome reporting hindered treatment outcome pooling. There were no comparative studies that evaluated observation only versus intervention in asymptomatic disease or strategies for monitoring treatment response. Conclusions: Evidence supporting optimal imaging modalities and early joint-preserving interventions remains limited and predominantly observational, underscoring the need for high-quality comparative studies with consistently defined core outcomes to guide clinical decision-making.

## 1. Introduction

Nontraumatic osteonecrosis of the femoral head (ONFH) is a progressive, debilitating disorder characterized by compromised blood supply to the femoral head, leading to osteocyte death, structural bone failure, and eventual articular collapse. If left untreated, ONFH frequently progresses to secondary hip osteoarthritis, often necessitating total hip arthroplasty (THA) at a relatively young age [1,2,3]. Approximately 10 to 20 thousand patients are diagnosed with osteonecrosis of the femoral head (ONFH) each year in the United States, with even higher prevalence reported in some Asian countries, and roughly 10% of all total hip arthroplasty patients have this diagnosis [1,2,3,4].

The pathophysiology of ONFH is multifactorial and incompletely understood [5,6]. Proposed mechanisms include intravascular thrombosis, extravascular compression, endothelial dysfunction, and impaired bone repair, all of which converge on reduced femoral head perfusion [1,2,3,4,5,6]. Common nontraumatic etiologies include systemic corticosteroid exposure, excessive alcohol consumption, hemoglobinopathies, autoimmune disease, solid organ transplantation, and idiopathic disease. Importantly, disease progression is strongly influenced by lesion size, location within the femoral head, and mechanical loading patterns, factors that complicate both prognostication and treatment selection [1,2,3,4,5,6].

Early-stage ONFH is often asymptomatic or minimally symptomatic, yet structural compromise may already be present [1,2,3,6]. Once subchondral fracture occurs, the likelihood of femoral head collapse rises sharply, and joint-preserving options become limited [1,2,3,6]. Consequently, accurate early diagnosis and precise staging are central to clinical decision-making. Several staging systems have been proposed, including the Ficat–Arlet, Steinberg, and Association Research Circulation Osseous (ARCO) classifications, with the ARCO system increasingly favored due to its incorporation of modern imaging findings and explicit distinction between precollapse and postcollapse disease [1,6].

Imaging plays a pivotal role across the disease spectrum [1,3,5,6]. Plain radiography remains widely used but lacks sensitivity for early disease [5,6]. Magnetic resonance imaging (MRI) is considered the reference standard for early detection and for delineating lesion extent, while computed tomography (CT) may better characterize subchondral fracture and early collapse [1,3,5,6]. However, uncertainty persists regarding the optimal imaging modality for specific clinical purposes, including differentiation of symptomatic versus asymptomatic disease and monitoring response to treatment. Moreover, advances in MRI and CT technology over the past decade have outpaced the evidence base guiding their clinical use in ONFH [1,3,6].

Management of ONFH varies widely across and within countries due to limited high-quality evidence, inconsistent disease-staging systems, and differences in cultural expectations, surgeon preferences, and resource availability [6]. In precollapse disease (ARCO stages I–II), joint-preserving interventions such as core decompression, biologic augmentation, bone grafting, and proximal femoral osteotomy aim to delay or prevent femoral head collapse [1,3,5,6]. In postcollapse disease (ARCO stage III and beyond), arthroplasty remains the preferred definitive treatment for most patients [1,3,5,6]. Despite decades of investigation, no single intervention has demonstrated clear superiority across patient subgroups or disease stages. Because no single treatment has been clearly shown to be superior at any given disease stage, surgeons often individualize care based on patient factors and extent of femoral head involvement [6]. These challenges highlight the need for evidence-based clinical practice guidelines to bring greater consistency and improve patient outcomes. To meet this need, the Association Research Circulation Osseous (ARCO), an international society devoted to the study of bone circulation, convened a workgroup of international experts in the management of ONFH to develop an evidence-based clinical practice guideline [7]. We conducted this systematic review on the diagnosis and treatment of nontraumatic ONFH to help the ARCO clinical practice guideline (CPG) workgroup develop recommendations for the evidence-based CPG on this topic. This systematic review is intended to serve as a resource for the ARCO CPG workgroup voting and does not contain methodology and results of the ARCO CPG workgroup voting or the final consensus-based guideline statements. The Key Questions (KQs) for this systematic review are as follows:

**KQ #1**: In patients undergoing diagnostic evaluation for ONFH, what imaging studies are most sensitive and specific for (a) diagnosis? (b) detecting subchondral fracture? (c) monitoring the effect of any intervention? (d) correlating symptomatic versus asymptomatic disease?

**KQ #2**: In patients who have ARCO stages I to II ONFH (without femoral head subchondral fracture or collapse): (a) What treatment is best at preventing femoral head subchondral fracture? (b) For asymptomatic patients, does treatment versus serial observation reduce the risk of femoral head subchondral fracture? (c) For symptomatic patients, should total hip arthroplasty (THA) be performed to reduce pain?

**KQ #3**: In patients who have ARCO stage 3 ONFH (with a femoral head fracture, with or without collapse), what surgical treatment (e.g., rotational osteotomy, hemiarthroplasty, surface replacement arthroplasty, or total hip arthroplasty) yields the best functional outcome for patients who have (a) an estimated lifespan less than 25 years? (older patients); (b) an estimated lifespan greater than 25 years? (younger patients).

## 2. Methods

This systematic review was conducted based on guidance in the U.S. Agency for Healthcare Research and Quality Methods Guide and the GRADE framework and reported using the Preferred Reporting Items for Systematic Reviews and Meta-Analyses (PRISMA), including the extension for Diagnostic Test Accuracy [8,9,10,11]. The protocol was prospectively registered in PROSPERO (CRD42024200381).

### 2.1. Study Selection Criteria

For this systematic review, we applied key question-specific inclusion and exclusion criteria (Table S1) to ensure relevance and methodological rigor [7]. For KQ1, we included human studies of adults (greater than 16 years) focused on diagnostic imaging test accuracy-related outcomes rather than treatment outcomes and excluded non-English full texts, case reports, interventions evaluated in only one small single-center study (less than 100 patients), and non-original data. For KQ2, we included studies of non-traumatic ONFH in ARCO Stages I to II that were human, prospective or retrospective, and enrolled greater than 15 patients, who had advanced imaging (CT or MRI) and ≥ two years of follow-up assessing radiographic progression and evaluating interventions that were tested in at least two independent eligible studies; standard exclusions applied. For KQ3, we included human studies of ARCO Stage III ONFH meeting similar methodological criteria (evidence levels 1 to 4, greater than 15 patients, uniform treatment, staging, and outcome data, ≥2-year follow-up) and applied the same exclusions.

### 2.2. Search Strategy and Screening

We conducted comprehensive searches in Medline (Ovid and PubMed), EMBASE, Web of Science Core Collection, SCOPUS, Global Index Medicus, and the Cochrane Library. A qualified medical librarian developed three unique search strategies, one for each KQ, following principles in the Peer Review of Electronic Search Strategies (PRESS) 2015 guideline [12]. The search strategies were reviewed and refined by the study team (Supplementary S1). The review team conducted dual independent reviews of titles/abstracts and full texts of potentially eligible studies on the cloud-based screening portal Rayyan. Additionally, we hand-searched reference lists of included studies and relevant systematic reviews. A final survey of all ARCO guideline workgroup members confirmed consensus on the completeness and adequacy of the literature search.

### 2.3. Data Extraction

Study-level data were extracted into Microsoft Excel sheets using templates based on the Template for Intervention Description and Replication (TIDieR) checklist, including first author, year of publication, digital identifier, descriptive data for study population, interventions, comparators, outcomes, and results [13].

### 2.4. Risk of Bias Assessment

The risk of bias (ROB) in included studies was assessed using the QUADAS-2 tool for studies of diagnostic test accuracy, the Cochrane ROB2 tool for randomized controlled trials (RCTs), and the ROBINS-I tool for non-randomized studies of interventions [14,15,16].

### 2.5. Data Analyses

We pooled data for a given intervention–comparator–outcome set of diagnostic imaging modalities when they were evaluated in two or more studies. For diagnostic test accuracy, we conducted meta-analyses using the bivariate, random effects model and the hierarchical summary receiver operating characteristics (sROC) model to jointly synthesize sensitivity and specificity across studies, accounting for within-study and between-study variability [17]. Summary estimates were presented with 95% confidence intervals, and heterogeneity was assessed by visual inspection of coupled forest plots and examination of model-derived variance components. For comparative effectiveness outcomes, we pooled risk ratios using a random-effects model with the Hartung–Knapp–Sidik–Jonkman (HKSJ) estimator to provide more robust inference in the presence of heterogeneity and small study effects [18]. We conducted analyses using R version 4.4.0 (R Core Team, 2024) or Stata 15 software (College Station, TX, USA; StataCorp LLC).

### 2.6. Strength of Evidence Assessment

The strength of evidence was assessed for each key outcome–intervention–comparator set using the GRADE framework [10]. Certainty of evidence was rated as high, moderate, low, or very low, with possible downgrades for risk of bias, consistency, directness, precision, and publication bias; and possible upgrades for large effect sizes, evidence of a dose–response relationship, and situations in which all plausible confounding would reduce a demonstrated effect. This approach ensured a systematic, transparent, and reproducible assessment of the certainty of evidence across all KQs. Certainty of evidence assessment was conducted only for outcomes drawn from direct comparisons with relevant intervention–comparator pairs relevant to KQs. We did not conduct certainty of evidence assessments for data from non-comparative (single-arm) studies.

## 3. Results

**Figure 1:**
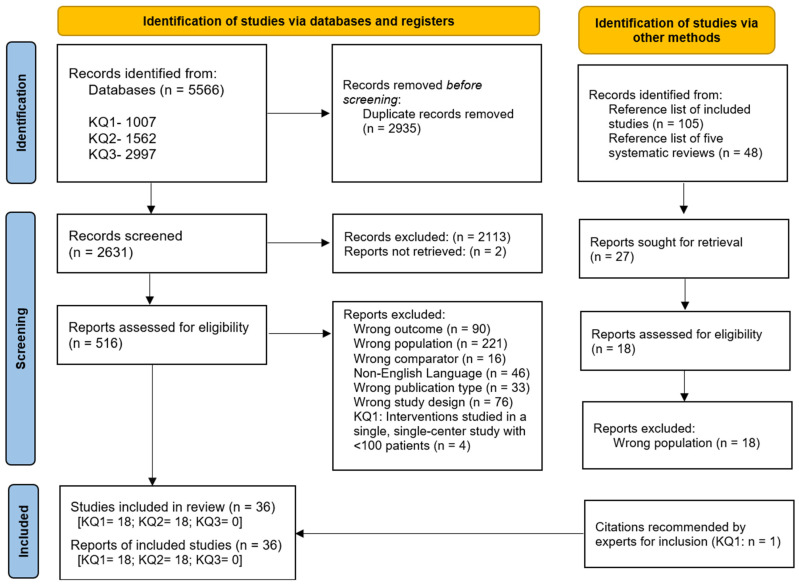
PRISMA Flow Diagram for Study Selection for Systematic Review: Diagnosis and Treatment of Osteonecrosis of Femoral Head. KQ: Key Question; Source: Page et al. [10]. This work is licensed under CC BY 4.0. To view a copy of this license, visit https://creativecommons.org/licenses/by/4.0/ (accessed on 17 February 2026).

From 5566 citations, we included 36 studies (Figure 1 and Figure S1), with 18 studies under KQ1 and 18 studies under KQ2 [19,20,21,22,23,24,25,26,27,28,29,30,31,32,33,34,35,36,37,38,39,40,41,42,43,44,45,46,47,48,49,50,51,52,53,54]. We found no eligible studies under KQ3. Study-level metadata and study characteristics are presented in Tables S2 and S3, and ROB assessments are in Figures S2–S4.

### 3.1. KQ1a: Modality for Diagnosis

For KQ1a, we included nine comparative studies from the United States, Korea, China, and India (sample sizes [n] = 20 to 440 hips), primarily used histology as the reference standard, and compared MRI, SPECT, bone scintigraphy (BS), and radiography [19,20,21,22,23,24,25,26,27]. Nearly all comparative imaging studies showed high ROB in patient selection owing to retrospective designs and case-enriched samples and mixed judgment in reference standard blinding, with several studies assessed as high or unclear ROB (Figure S2).

**Figure 2:**
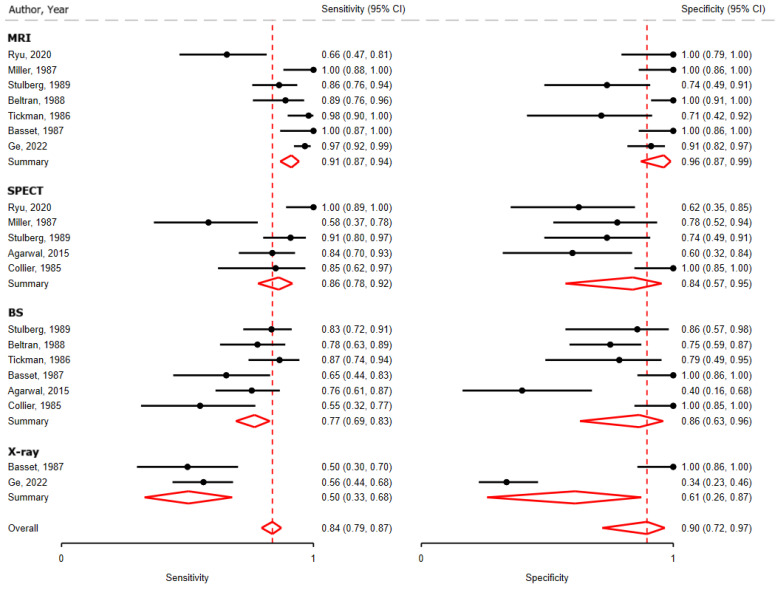
Forest Plots and Meta-Analysis for Key Question 1: Diagnosis and Evaluation of Osteonecrosis of the Femoral Head. BS—bone scintigraphy; MRI—magnetic resonance imaging; SPECT—single-photon emission computed tomography.

Magnetic resonance imaging demonstrated the highest overall diagnostic performance (Figure 2 and Table 1; very low-grade evidence). Among all modalities evaluated, MRI had the greatest pooled sensitivity (0.91; 95% confidence interval (CI), 0.87 to 0.94) and pooled specificity (0.96; 95% CI, 0.87 to 0.99). Standard radiography showed the poorest pooled sensitivity (0.50; 95% CI, 0.33 to 0.68) and specificity (0.61; 95% CI, 0.26 to 0.87). The sROC plot indicated imprecision in estimates (Figure S5).

### 3.2. KQ1b: Detection of Subchondral Fracture

For KQ1b (detection of subchondral fracture), we included four retrospective comparative studies (n = 45 to 1001 hips), one from China, one from Korea, and two from the USA, comparing frog-leg lateral and AP radiographs, digital radiography, MRI, and computed tomography (CT), with outcomes focused on sensitivity, specificity, predictive values, and accuracy [28,29,30,54]. All studies were assessed as high ROB in patient selection and flow/timing, reflecting heterogeneous imaging intervals and inconsistent application of reference standards (Figure S2).

A study reported that for detecting subchondral fractures, CT had the highest diagnostic yield, followed by MRI and X-ray (Table 1; very low-grade evidence) [29]. Another study concluded that 3-T MRI was not inferior to CT in detecting cases of ARCO stage III ONFH [54]. There were two retrospective studies that reported conflicting findings for the benefit of adding a frog-leg image in detecting subchondral fracture compared with a standard image alone, with one study favoring incremental diagnostic yield and another study reporting no difference in diagnostic yield (Table 1; very low-grade evidence) [28,30].

### 3.3. KQ1c: Monitoring Treatment Response

For KQ1c (monitoring treatment response), we identified three studies (n = 13 to 90 hips), one each from Austria, Greece, and the USA, evaluating MRI versus bone scintigraphy for serial assessment of necrosis volume after intervention, all relying on histology or MRI-based volumetry [27,31,32]. All three studies were assessed as low ROB in the index test, but high ROB in patient selection and flow/timing, consistent with retrospective cohorts and lack of standardized follow-up imaging, while applicability concerns remained minimal.

There was one comparative study that reported that MRI has better diagnostic performance than BS to monitor the effect of treatment in terms of detecting the extent of necrotic tissue (Table 1; very low-grade evidence) [27]. There were two single-arm studies that also demonstrated the utility of MRI in postoperative surveillance after core decompression, vascularized bone graft, and total arthroplasty [31,32].

### 3.4. KQ1d: Distinguishing Symptomatic from Asymptomatic ONFH

For KQ1d, we included three observational MRI-based studies (n = 37 to 123 hips), one each from Japan, China, and Korea, that examined correlations between bone marrow edema, joint effusion, and the presence of hip pain [33,34,35]. All three studies were assessed as unclear or high ROB in patient selection and in reference standard, as symptom assessment lacked blinding and relied on clinical self-report.

To differentiate symptomatic from asymptomatic hips, both bone marrow edema (BME) had higher pooled specificity for pain (0.98, 95% CI, 0.79 to 1.00) and grade 2+ effusion (0.80, 95% CI, 0.64 to 0.90) also had good specificity for pain (but lower than BME; Figure S6). The BME also had a greater positive likelihood ratio and diagnostic odds ratio compared with grade 2+ effusion (Table 1 and Table S4, Figure S7), suggesting greater accuracy of BME for differentiating symptomatic from asymptomatic nontraumatic ONFH (Table 1; moderate-grade evidence). Additionally, one study reported a greater probability of BME with increasing severity of pain and that in a logistic regression model adjusting for both indicators, only BME remained a significant predictor of pain [35].

### 3.5. KQ2a: Optimal Management of ARCO Stages I to II ONFH

We included 14 studies under KQ2a evaluating head-preserving interventions for early-stage ONFH with radiographic progression to collapse as the outcome of interest [36,37,38,39,40,41,42,43,44,45,46,47,48,49]. There were three studies that compared core decompression (CD) with and without bone marrow concentrate (BMC): two randomized controlled trials from Belgium and Germany (n = 24 hips and n = 25 hips; follow-up 24 to 60 months) and one U.S. retrospective comparative cohort assessing CD plus BMC versus CD alone [36,37,38]. There were three studies that compared vascularized fibular grafting (VBG) versus non-vascularized bone grafting (non-VBG): one randomized controlled trial from China (n = 46 versus n = 45 hips, respectively) and two retrospective matched cohort studies from Korea and the United States (n = 10 versus 10 hips respectively; n = 35 versus 35 hips, respectively), all reporting collapse outcomes at 50 to 60 months of follow-up [39,40,41]. Additional single-arm series (k = 4 studies) described outcomes of VBG without comparator groups, with sample sizes ranging from 17 to 39 hips and follow-up extending to 91 months [42,43,44,45]. There were two single-arm studies that evaluated osteotomy techniques, including transtrochanteric rotational osteotomy (n = 36 hips) and intertrochanteric curved varus osteotomy (n = 30 hips), with follow-up of 75 to 84 months [46,47]. A comparative cohort from Germany evaluated intertrochanteric osteotomy versus CD [48]. An Indian cohort compared CD plus non-VBG versus CD alone [49].

Across the three RCTs, ROB-2 assessments (Figure S3) showed high ROB, primarily due to issues in the randomization process and missing outcome data, with additional concerns in deviations from intended interventions [36,37,39]. The observational, comparative, and single-arm studies demonstrated serious or critical ROB in ROBINS-I, driven mainly by confounding and participant selection, though most studies had low risk in outcome measurement and reporting domains.

**Figure 3:**
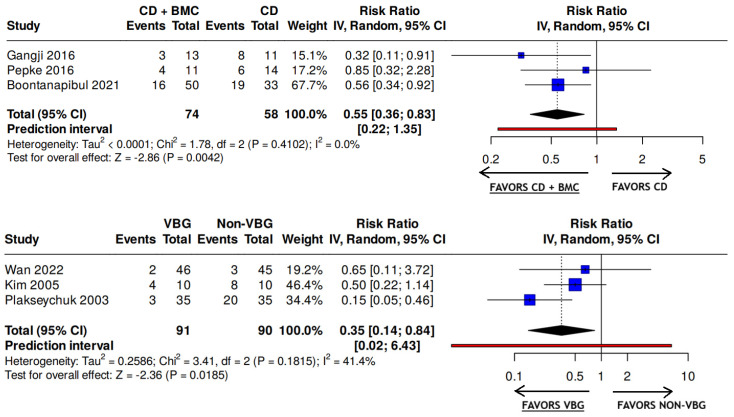
Forest Plots and Meta-Analysis for Rate of Collapse After Surgical Treatment of ARCO Stages I–II Osteonecrosis of the Femoral Head. BMC—bone marrow concentrate; CD—core decompression; VBG—vascular bone graft.

Across three studies (n = 132 hips; two RCTs and one retrospective study; Figure 3) evaluating CD with versus without BMC, the pooled risk of femoral head collapse was lower in the CD plus BMC group (31.1%) compared with CD alone (56.9%); the overall certainty of evidence was very low (Table 2) [36,37,38]. Meta-analysis demonstrated a pooled risk ratio of 0.55 (95% CI, 0.36 to 0.83), indicating a relative reduction in collapse risk (Figure 3). Similarly, pooling data from three studies (n = 181 hips; one RCT and two retrospective studies; Figure 3) comparing VBG with non-VBG showed a markedly lower collapse rate with VBG (9.9%) versus non-VBG (34.4%), yielding a pooled risk ratio of 0.35 (95% CI, 0.14 to 0.84), with very low certainty of evidence (Table 2) [39,40,41].

Osteotomies were evaluated in three eligible single-arm studies with no comparator group [46,47,48]. The incidence of collapse was 11% (95% CI, 0 to 35%) over a follow-up period of greater than 10 to 20 years postoperatively. Due to the lack of a comparator group, the strength of evidence was not rated. Of note, all three studies used different techniques for osteotomy chosen based on clinical context. There was one study that compared CD plus non-VBG versus CD alone and reported no significant difference in rate of collapse [49].

### 3.6. KQ2b: Observation Versus Treatment for ARCO Stages I to II ONFH

For KQ2b, we found no studies directly comparing observation versus treatment, and we included four cohort studies (n = 68 to 121 hips; follow-up six to 168 months) that described the natural history of untreated asymptomatic ONFH, reporting progression to symptoms for observation alone [50,51,52,53]. All four studies were assessed at critical ROB owing to the single-arm design and no comparator (i.e., treated) group. Kang et al. (n = 68) reported 56% becoming symptomatic at a mean of 2.3 years; larger and laterally located lesions were significant predictors [51]. Nam et al. (n = 105) found 59% became symptomatic at approximately 25 months; extent and size strongly predicted progression [53]. In systemic lupus patients (n = 121), Hernigou et al. reported 95% of stage I and 100% of stage II hips developed pain within two to three years [50]. Min et al. (n = 81) found 32% collapsed at a mean of four years; large, laterally located (C2) lesions carried the highest risk [52].

We found no studies that met our eligibility criteria to answer KQ2c and KQ3. Therefore, the only means to address these questions for a guideline would default to consensus opinion by subject matter experts.

## 4. Discussion

This systematic review offers an integrated assessment of diagnostic and therapeutic evidence for nontraumatic ONFH, with the explicit goal of informing development of an evidence-based ARCO CPG. Several key findings emerge that have important implications for clinical practice across varied settings, research prioritization, and guideline formulation.

### 4.1. Diagnostic Imaging: Clinical Interpretation and Implications

Across comparative diagnostic accuracy studies, MRI demonstrated the highest sensitivity and specificity for early detection of ONFH. This finding is consistent with the biological basis of ONFH, in which early marrow and trabecular changes precede radiographic abnormalities. Notably, several studies included in our meta-analyses are old and may underestimate the performance of MRI in current times, given the advancement in MRI technology and diagnostic criteria over the years [55]. More recent, well-designed comparative studies may help improve certainty of evidence ratings to rise above very low grade. Beyond diagnosis, MRI findings—most notably bone marrow edema—showed strong associations with symptomatic disease in precollapse ONFH. These observations reinforce the clinical utility of MRI not only as a diagnostic tool, but also as a means of phenotyping disease activity and identifying patients at higher risk for pain and progression [54,55].

The CT and MRI both outperformed plain radiography in detecting subchondral fractures, although the certainty of evidence was very low. From a clinical perspective, this highlights an important diagnostic inflection point: once subchondral fracture occurs, the probability of femoral head collapse increases substantially, often altering management from joint-preserving strategies to arthroplasty [55]. The lack of high-quality comparative data limits firm recommendations regarding optimal modality selection, suggesting that imaging choice should be individualized based on availability, expertise, radiation exposure considerations, and clinical suspicion [54,55,56].

Our findings are highly applicable in modern orthopedic practice, where MRI is widely used and increasingly accessible [55]. However, applicability varies across health systems, as differences in MRI protocols, radiologic expertise, and imaging availability may influence diagnostic yield. Similarly, both CT and MRI may be used to detect subchondral fracture, though selection of modality may depend on local considerations regarding availability of technology and expertise, radiation exposure, and cost [54,56].

Evidence regarding imaging for monitoring treatment response was notably sparse. While MRI appears capable of quantifying necrotic volume and postoperative changes, thresholds for clinically meaningful change remain undefined. This gap limits the ability to use imaging as a surrogate endpoint in both clinical practice and research, underscoring the need for standardized imaging-based outcome measures.

### 4.2. Treatment of Precollapse ONFH: Interpreting Comparative Evidence

The therapeutic findings also align with and expand upon earlier systematic reviews. For early-stage ONFH (ARCO stages I–II), core decompression (CD) remains the most frequently studied intervention. Prior reviews emphasized the promise of CD in ONFH yet noted variability in outcomes, pooled data across disparate interventions, and did not report findings exclusively for precollapse ONFH (i.e., ARCO stages I or II) [57,58,59]. Our meta-analyses, incorporating more recent comparative studies, support those observations and provide evidence specifically for precollapse ONFH, suggesting improved radiographic outcomes when CD is combined with BMC. This is consistent with emerging biologic literature, which proposes enhanced reparative potential when CD is augmented with autologous cell-based therapies [60]. However, the certainty of evidence remains very low due to small sample sizes, risk of bias, and heterogeneity in patient selection and procedural technique.

Similarly, earlier reviews of vascularized fibular grafting and osteotomy have highlighted their potential role in experienced centers [61,62,63]. Our meta-analysis provides evidence specifically for precollapse ONFH, which should be contextualized with procedural complexity, surgeon expertise, and perioperative resources that might influence the feasibility and success of these interventions across practice environments. Notably, evidence for osteotomies comes from very few, non-comparative case series; however, the rate of postoperative collapse for osteotomies in these studies appears to be lower than that for other procedures. This finding suggests the need for large, well-designed, comparative effectiveness studies to evaluate whether osteotomies may be superior to other procedural options for treating precollapse ONFH [46,47,48].

Importantly, inconsistent outcome definitions across studies, particularly regarding collapse, progression, and functional recovery, and highly variable length of follow-up across studies substantially hindered pooling and interpretation. This limitation reflects a broader challenge in ONFH research and directly constrains the strength of guideline recommendations. There is a dire need to develop a standardized core outcomes set and a rigorous reporting framework for future RCTs and clinical research on ONFH to aid future pooling/synthesis of high-certainty evidence.

### 4.3. Natural History and the Challenge of Asymptomatic Disease

The natural history of asymptomatic ONFH remains poorly defined. Observational cohorts suggest that a substantial proportion of asymptomatic hips progress to pain or collapse, particularly in the presence of large or laterally located lesions. However, the absence of comparative studies evaluating observation versus early intervention prevents definitive conclusions regarding the benefit of prophylactic treatment. This uncertainty poses a common clinical dilemma and highlights an area where expert consensus, rather than evidence, may guide practice.

The natural history data for asymptomatic ONFH further underscores the importance of contextual factors. Progression to symptomatic disease or collapse varied widely across studies, reflecting differences in lesion size, location, comorbidities, and patient populations. These contextual variables are essential when interpreting progression risk, particularly in settings with differing prevalence of corticosteroid use, alcohol exposure, or systemic inflammatory conditions [1,2,3,4,5]. In advanced-stage ONFH, findings from this review reaffirm the variability in reported outcomes across joint-preserving procedures. Applicability of these interventions is closely tied to local expertise, patient selection practices, and operative resources, all of which shape real-world outcomes and should be considered when interpreting the comparative data.

### 4.4. Methodological Limitations of the Evidence Base

The existing evidence base for the diagnosis and treatment of ONFH has several important potential limitations that constrain the certainty of evidence. Many studies are small, single-center case series with heterogeneous populations, inconsistent staging criteria, and variable use of advanced imaging, making comparisons across studies difficult. Few investigations include long-term follow-up, and outcomes are often inconsistently defined, particularly with respect to radiographic progression and functional recovery. Treatment cohorts frequently lack uniformity in intervention protocols, and many studies do not adequately control potential confounding due to key prognostic factors such as lesion size, location, or etiology. Furthermore, studies often focused on clinically heterogeneous, mixed populations (e.g., mixing ARCO stages I to III disease), limiting evidence synthesis and generalizability of findings. This was one of the reasons why we did not find any eligible studies for supporting KQ 2c and KQ 3. Additionally, the predominance of observational designs, coupled with inadequate control for confounding and incomplete outcome reporting, limits the strength of causal inferences. These methodological weaknesses directly influenced GRADE certainty ratings, which were low or very low for most outcomes. As a result, even when pooled estimates suggest benefit, confidence in the magnitude and durability of effect remains limited.

### 4.5. Strengths and Limitations of the Review Process

This review’s strengths include the use of comprehensive, librarian-assisted search strategies; systematic evaluation of certainty of evidence using the GRADE framework; the rigor assured by leadership from highly experienced systematic review methodologists; and the consensus building using input from a large group of international physicians (eight countries) with subject expertise. Our use of hierarchical models for diagnostic accuracy and random-effects HKSJ methods for comparative outcomes further enhances analytical robustness. However, limitations include variability in study designs and reporting, heterogeneity in imaging and outcome measures, and the predominance of observational evidence for several key questions. These factors may reduce precision and limit the generalizability of some findings.

### 4.6. Implications for Guidelines and Future Research

Future research priorities should include well-designed, multicenter comparative studies with standardized staging, imaging protocols, and clearly defined outcomes. Development of a standardized core outcome set including radiographic, clinical, and patient-reported outcomes with long-term follow-up would substantially enhance comparability across trials and improve the evidentiary foundation for future guidelines.

From a guideline-development perspective, these findings highlight both areas of relative consensus and substantial evidence gaps. MRI can be recommended as the preferred modality for early diagnosis and assessment of symptomatic disease, while recommendations regarding specific joint-preserving treatments should be conditional and context-dependent.

## 5. Conclusions

This systematic review synthesizes the available evidence on diagnostic imaging and treatment outcomes for nontraumatic ONFH to inform the development of an ARCO clinical practice guideline. Among different modalities, MRI demonstrated the most consistent diagnostic accuracy, reliably detecting early ONFH and identifying bone marrow edema and joint effusion associated with symptomatic disease. Limited comparative data suggested CT more frequently identified subchondral fractures than radiography or MRI. Evidence on imaging for monitoring treatment response was sparse, with MRI being the only modality repeatedly shown to quantify lesion extent, though thresholds for meaningful change remain undefined. For early-stage ONFH (ARCO stages I and II), CD was the most studied intervention; small comparative studies suggested lower collapse rates when combined with BMC. Similarly, very low-grade evidence suggests lower rates of collapse with VBG compared with non-VBG. Other joint-preserving procedures were supported only by heterogeneous, single-center observational studies, limiting inference. There was no comparative study that evaluated observation only versus intervention in asymptomatic disease or strategies for monitoring treatment response. Overall, substantial methodological heterogeneity and predominant reliance on observational designs highlight the need for high-quality research with standardized reporting measures to enable comparative analysis for research in ONFH.

## Figures and Tables

**Table 1:** Strength of Evidence Assessments for Key Question 1 by GRADE Criteria for Systematic Review: Diagnosis and Treatment of Nontraumatic Osteonecrosis of Femoral Head.

Key Question (KQ): Statement	K = Number of Studies (n = Sample Size) Risk of Bias (ROB)	Summary of Findings	Consistency Precision Directness Publication Bias Other Considerations	Grade
KQ1a: MRI is the preferred modality for diagnosis of ONFH due to higher sensitivity and specificity compared with CT, SPECT, bone scintigraphy, or X-ray	K = 9 studies (n = 933 hips) 9 high ROB	Pooled sensitivity (Sn) and pooled specificity (Sp) shown in Figure 2, for: MRI– Sn = 0.91 (95% CI, 0.87–0.94), Sp = 0.96 (95% CI, 0.87–0.99); SPECT– Sn = 0.86 (95% CI, 0.78–0.92), Sp = 0.84 (95% CI, 0.57–0.95); Bone scintigraphy– Sn = 0.77 (95% CI, 0.69–0.83), Sp = 0.86 (95% CI, 0.63–0.96); X-ray– Sn = 0.50 (95% CI, 0.33–0.68), Sp = 0.61 (95% CI, 0.26–0.87).	Serious Serious Not serious Not serious None	Very low ⊕◯◯◯
KQ1b: CT or MRI are preferred over plain radiography for detecting subchondral fractures.	K = 2 studies (n = 273 hips) 2 high ROB	One study reported that CT detected more fractures than 1.5-T MRI or X-ray. Compared with CT, 1.5-T MRI has a sensitivity and specificity of 38 and 100%, and unenhanced radiography has a sensitivity and specificity of 71 and 97%. One study reported that 3-T MRI was not inferior to CT.	Serious Serious Not serious Not serious None	Very low ⊕◯◯◯
KQ1b: There is conflicting evidence to justify the benefit of adding frog-leg lateral radiograph to standard antero-posterior radiograph in detecting subchondral fracture.	K = 2 studies (n = 1403 hips) 2 high ROB	One study comparing frog-lateral vs. AP radiographs (n = 1001 hips) found that the combined radiographic approach demonstrated the highest diagnostic performance, with sensitivity 87% and specificity 100%. Another study found no incremental diagnostic value from adding the frog-leg image.	Serious Serious Not serious Not serious None	Very low ⊕◯◯◯
KQ1c: MRI has better diagnostic performance than BS, to monitor the effect of treatment in terms of detecting the extent of necrotic tissue	K = 1 study (n = 45 hips) 1 high ROB	One study showed superior sensitivity and specificity of MRI (86 and 98%, respectively) compared with BS (79 and 71%, respectively) to detect necrotic tissue.	Cannot assess Serious Not serious Not serious None	Very low ⊕◯◯◯
KQ1d: In precollapse nontraumatic ONFH, bone marrow edema and grade 2+ effusion on MRI can help differentiate symptomatic from asymptomatic hip	K = 3 studies (n = 270 hips) 2 low ROB and 1 unclear ROB	MRI signs have good, pooled specificity for pain: MRI—0.98 (95% CI, 0.88, 1.00) Effusion—0.80 (95% CI, 0.64, 0.90)	Not serious Serious * Not serious Not serious Upgraded for large estimate (pooled specificity)	Moderate ⊕⊕⊕◯
KQ1d: In precollapse nontraumatic ONFH, on MRI, bone marrow edema is more accurate than grade 2+ effusion to differentiate symptomatic (painful) from asymptomatic hip	K = 3 studies (n = 270 hips) 2 low ROB and 1 unclear ROB	Pooled diagnostic odds ratio (OR): BME—OR = 35.68 (95% CI, 4.50, 283.08) Effusion—OR = 10.14 (95% CI, 5.57, 21.63) Pooled positive likelihood ratio: BME—16.21 (95% CI, 3.50, 75.13) Effusion—3.26 (95% CI, 2.06, 5.16)	Serious Serious * Not serious Not serious Upgraded for large estimate and dose–response association (greater likelihood of BME for greater degree of pain in precollapse ONFH)	Moderate ⊕⊕⊕◯

* Downgraded twice for precision, for wide confidence interval and small total sample size. BME—bone marrow edema; BS—bone scintigraphy; CT—computed tomography; MRI—magnetic resonance imaging; OR—odds ratio; ROB—risk of bias; T—Tesla.

**Table 2:** Strength of Evidence Assessments for Key Question 2 by GRADE Criteria for Systematic Review: Diagnosis and Treatment of Nontraumatic Osteonecrosis of the Femoral Head.

Key Question (KQ): Statement	K = Number of Studies (n = Sample Size) Risk of Bias (ROB)	Summary of Findings	Consistency Precision Directness Publication Bias Other Considerations	Grade
KQ2a: For patients undergoing core decompression, adding bone marrow concentrate to the procedure may reduce the risk of femoral head collapse	K = 3 studies (n = 132 hips) 1 moderate ROB RCT 1 high ROB RCT 1 high ROB NRSI	Risk of collapse in: CD + BMC = 31.08% and CD = 56.90% Pooled RR (Figure 3) = 0.55 (95% CI, 0.36, 0.83)	Serious Serious Not serious Not serious None	Very low ⊕◯◯◯
KQ2a: For patients undergoing bone grafting, compared with non-vascularized bone graft, vascularized bone graft may reduce the risk of femoral head collapse	K = 3 studies (n = 181 hips) 2 high ROB RCTs 1 high ROB NRSI	Risk of collapse in: VBG = 9.89% and non-VBG = 34.44% Pooled RR (Figure 3) = 0.35 (95% CI, 0.14, 0.84)	Serious Serious Not serious Not serious None	Very low ⊕◯◯◯

BMC—bone marrow concentrate; CD—core decompression; KQ—key question; NRSI—nonrandomized study of intervention; RCT—randomized controlled trial; ROB—risk of bias; RR—relative risk ratio; VBG—vascularized bone graft.

## Data Availability

The original contributions presented in this study are included in the article/Supplementary Material. Further inquiries can be directed to the corresponding author.

## Supplementary Materials

The following supporting information can be downloaded at: https://www.mdpi.com/article/10.3390/medsci14010107/s1, Supplementary S1: Search Strategy and Screening: Supplemental Table S1. Study Selection Criteria for Systematic Review: Diagnosis and Treatment of Osteonecrosis of Femoral Head, Supplemental Figure S1 PRISMA Flow Diagram for Study Selection in Primary Searches; Supplementary S2. Description of Population, Intervention, Comparator, and Outcomes in Included Studies: Supplemental Table S2 Characteristic features of studies that were used in PICO1 recommendations, Supplemental Table S3 Characteristic features of studies that were used in PICO2 recommendations; Supplementary S3. Risk of Bias Assessments: Supplemental Figure S2 Risk of bias assessment using QUADAS-2 tool for studies under Key Question 1, Supplemental Figure S3 Risk of bias assessment using the Cochrane RoB2.0 tool for RCT studies under Key Question 2, Supplemental Figure S4 Risk of bias assessment using the ROBINS-I tool for non-RCT studies under Key Question 2; Supplementary S4. Meta-analyses: Supplemental Figure S5 Summary Receiver Operating Characteristics Plot for Diagnostic Test Accuracy Meta-Analysis for Different Modalities to Diagnose Osteonecrosis of Femoral Head, Supplemental Figure S6 Pooled Specificity and Sensitivity Estimates for KQ1d: Differentiating Asymptomatic versus Symptomatic Nontraumatic ONFH, Supplemental Figure S7 Positive Likelihood Ratios for MRI Signs to Differentiate Symptomatic versus Asymptomatic Nontraumatic ONFH, Supplemental Table S4. Diagnostic Odds Ratios for MRI signs to Differentiate Between Symptomatic Versus Asymptomatic Nontraumatic ONFH.
