# Supplementary material for: Diagnosis and Treatment of Nontraumatic Osteonecrosis of the Femoral Head: A Systematic Review and Meta-Analyses for the ARCO Clinical Practice Guideline Development Workgroup
Source: Med Sci (Basel). 2026 Feb 23;14(1):107. doi: 10.3390/medsci14010107 (PMC13027876; doi:10.3390/medsci14010107)
Supplement: Supplementary file 1 [file medsci-14-00107-s001.zip › Supplement_Final draft_ARCO SR MA 1.pdf]

**Manuscript Title**: Diagnosis and Treatment of Nontraumatic Osteonecrosis of Femoral Head: A Systematic Review and Meta-Analyses for Development of the ARCO Clinical Practice Guideline.

**Online Supplement**

**S1. Search Strategy and Screening**

Search Strategies

*Key Question 1: Diagnostic Imaging*

| Ovid MEDLINE(R) ALL | |
|---|---|
| 1 | osteonecrosis/ |
| 2 | exp Osteoradionecrosis/ |
| 3 | 1 or 2 |
| 4 | exp Femur Head/ |
| 5 | 3 and 4 |
| 6 | exp Femur Head Necrosis/ |
| 7 | ((femur or femoral) adj2 head).tw,kw. |
| 8 | ((avascular or aseptic or ischemic) adj2 (necrosis or necroses)).tw,kw. |
| 9 | (osteonecrosis or osteonecroses or osteoradionecrosis or osteoradionecroses).tw,kw. |
| 10 | 8 or 9 |
| 11 | 7 and 10 |
| 12 | onfh.tw,kw. |
| 13 | 5 or 6 or 11 or 12 |
| 14 | exp Diagnostic Imaging/ |
| 15 | ((diagnostic or "magnetic resonance" or medical) adj2 imag*).tw,kw. |
| 16 | "diagnostic imaging".fs. 1479543 |

| | |
|---|---|
| 17 | (mri* or tomograph* or radiograph* or ultrasonograph* or x-ray).tw,kw. |
| 18 | ((ct or cat) adj2 scan*).tw,kw. |
| 19 | 14 or 15 or 16 or 17 or 18 |
| 20 | exp "Sensitivity and Specificity"/ |
| 21 | sensitivity.tw,kw. |
| 22 | specificity.tw,kw. |
| 23 | ((pre-test or pretest) adj probability).tw,kw. |
| 24 | post-test probability.tw,kw. |
| 25 | predictive value*.tw,kw. |
| 26 | likelihood ratio*.tw,kw. |
| 27 | diagnostic accuracy.tw,kw. |
| 28 | 20 or 21 or 22 or 23 or 24 or 25 or 26 or 27 |
| 29 | 13 and 19 and 28 |
| 30 | exp Animals/ not exp Humans/ |
| 31 | 29 not 30 |
| 32 | exp adult/ |
| 33 | exp child/ or exp infant/ or exp adolescent/ |
| 34 | 33 not 32 |
| 35 | 31 not 34 |
| 36 | "case report".ti. |
| 37 | (letter or commentary or editorial).pt. |
| 38 | 36 or 37 |
| 39 | 35 not 38 |

| PubMed |
| --- |
| (((((((("Osteonecrosis"[Mesh:NoExp]) OR "Osteoradionecrosis"[Mesh]) AND ("Femur Head"[Mesh])) OR ("Femur Head Necrosis"[Mesh])) OR (("femur head"[tiab] OR "femoral head"[tiab]) AND (("avascular necrosis"[tiab] OR "avascular necrosis"[tiab] OR "ischemic necrosis"[tiab] OR "avascular necroses"[tiab] OR "avascular necroses"[tiab] OR "ischemic necroses"[tiab]) OR (osteonecrosis[tiab] OR osteonecroses[tiab] OR osteoradionecrosis[tiab] OR osteoradionecroses[tiab]))))) OR (onfh[tiab])) AND ((((("Diagnostic Imaging"[Mesh]) OR ("diagnostic imag*"[tiab] OR "magnetic resonance imag*"[tiab] OR "medical imag*"[tiab])) OR ("diagnostic imaging" [Subheading])) OR (mri[tiab] OR tomograph*[tiab] OR radiograph*[tiab] OR ultrasonograph*[tiab] OR x-ray[tiab])) OR ("ct scan"[tiab] OR "cat scan"[tiab]))) AND (((((((("Sensitivity and Specificity"[Mesh]) OR (sensitivity[tiab])) OR (specificity[tiab])) OR ("pre-test probability"[tiab] OR "pretest probability"[tiab])) OR ("post-test probability"[tiab])) OR ("predictive value*"[tiab])) OR ("likelihood ratio*"[tiab])) OR ("diagnostic accuracy"[tiab]))) NOT ("animals"[Mesh] NOT "humans"[Mesh])) NOT (("child"[Mesh] OR "infant"[Mesh] OR "adolescent"[Mesh]) NOT ("adult"[Mesh])) |
| Embase Classic+Embase (via Ovid) |
| 1 bone necrosis/<br>2 exp osteoradionecrosis/<br>3 1 or 2<br>4 exp femoral head/<br>5 3 and 4<br>6 exp femur head necrosis/<br>7 ((femur or femoral) adj2 head).tw,kw.<br>8 ((avascular or aseptic or ischemic) adj2 (necrosis or necroses)).tw,kw.<br>9 (osteonecrosis or osteonecroses or osteoradionecrosis or osteoradionecroses).tw,kw.<br>10 8 or 9<br>11 7 and 10 |

| | |
|---|---|
| 12 | onfh.tw,kw. |
| 13 | 5 or 6 or 11 or 12 |
| 14 | exp diagnostic imaging/ |
| 15 | ((diagnostic or "magnetic resonance" or medical) adj2 imag*).tw,kw. |
| 16 | (mri* or tomograph* or radiograph* or ultrasonograph* or x-ray).tw,kw. |
| 17 | ((ct or cat) adj2 scan*).tw,kw. |
| 18 | 14 or 15 or 16 or 17 |
| 19 | exp "sensitivity and specificity"/ |
| 20 | sensitivity.tw,kw. |
| 21 | specificity.tw,kw. |
| 22 | ((pre-test or pretest) adj probability).tw,kw. |
| 23 | post-test probability.tw,kw. |
| 24 | predictive value*.tw,kw. |
| 25 | likelihood ratio*.tw,kw. |
| 26 | diagnostic accuracy.tw,kw. |
| 27 | 19 or 20 or 21 or 22 or 23 or 24 or 25 or 26 |
| 28 | 13 and 18 and 27 |
| 29 | exp Animal/ not exp Human/ |
| 30 | 28 not 29 |
| 31 | exp adult/ |
| 32 | exp child/ or exp infant/ or exp adolescent/ |
| 33 | 32 not 31 |
| 34 | 30 not 33 |
| 35 | (letter or commentary or editorial).pt. |
| 36 | 34 not 35 |

| |
|---|
| Web of Science (Core Collection: SCI-EXPANDED, SSCI, AHCI, CPCI-S, CPCI-SSH, BKCI-SSH, ESCI, CCR-EXPANDED, IC) |
| TS=(((("femur head" OR "femoral head") NEAR/3 (osteonecrosis OR osteonecroses OR necrosis or necroses) OR osteoradionecrosis OR osteoradionecroses) OR ONFH) AND (((diagnostic OR "magnetic resonance" OR medical) NEAR/2 image) OR MRI OR tomograph* OR radiograph* OR ultrasonograph* OR x-ray OR "CT scan" OR "CAT scan" ) AND (sensitivity OR specificity OR (pretest NEAR/2 probability) OR "post test probability" OR "predictive value" OR "likelihood ratio" OR "diagnostic accuracy")) |
| Scopus |
| ((((INDEXTERMS("femur head necrosis")) OR (INDEXTERMS("Osteoradionecrosis" OR "Bone Necrosis" OR "Osteonecrosis") AND INDEXTERMS("Femur Head" OR "Femoral Head")) OR ((TITLE-ABS-KEY(((femur OR femoral) W/2 head))) AND (TITLE-ABS-KEY(((avascular OR aseptic OR ischemic) W/2 (necrosis OR necroses)) OR osteonecrosis OR osteonecroses OR osteoradionecrosis OR osteoradionecroses)) OR TITLE-ABS (ONFH))) AND ((INDEXTERMS("Diagnostic Imaging")) OR (TITLE-ABS((diagnostic NEAR/2 imag*) OR ("magnetic resonance" NEAR/2 imag*) OR (medical NEAR/2 imag*) OR mri* OR tomograph* OR radiograph* OR ultrasonograph* OR x-ray OR (ct NEAR/2 scan*) OR (cat NEAR/2 scan*)))) AND ((INDEXTERMS("Sensitivity and Specificity")) OR (TITLE-ABS(sensitivity OR specificity OR "pre-test probability" OR "pretest probability" OR "post-test probability" OR "predictive value" OR "likelihood ratio" OR "diagnostic accuracy")))) AND NOT ((INDEXTERMS(animal OR animals)) AND NOT (INDEXTERMS(human or humans)))) AND NOT ((INDEXTERMS(Child OR infant OR adolescent)) AND NOT (INDEXTERMS(adult))) |
| Global Index Medicus |
| **This search 3 results (lacks the sensitivity/diagnostic piece)** |

| |
|---|
| ((((mh:(Osteoradionecrosis)) or mh:(Osteonecrosis)) OR (mh:(Femur Head Necrosis))) OR (tw:(osteonecrosis OR osteonecroses OR osteoradionecrosis OR osteoradionecroses)) AND (tw:(imaging OR magnetic resonance OR MRI OR tomograph* OR radiograph* OR ultrasonograph* OR x-ray OR CT scan OR CAT scan))) AND (mh:(Sensitivity and Specificity) OR (tw:(sensitivity OR specificity OR pretest OR post-test OR predictive value* OR likelihood ratio* OR diagnostic))) <br><br> **Zero results (includes sensitivity/diagnostic piece)** <br><br> (((mh:(Osteoradionecrosis)) OR (mh:(Femur Head Necrosis)) AND (tw:(osteonecrosis OR osteonecroses OR osteoradionecrosis OR osteoradionecroses)) AND (tw:(imaging OR magnetic resonance OR MRI OR tomograph* OR radiograph* OR ultrasonograph* OR x-ray OR CT scan OR CAT scan))) AND (mh:(Sensitivity and Specificity) OR (tw:(sensitivity OR specificity OR pretest OR post-test OR predictive value* OR likelihood ratio* OR diagnostic))) |
| Cochrane Library (via Wiley) |
| #1 MeSH descriptor: [Osteonecrosis] this term only <br> #2 MeSH descriptor: [Osteoradionecrosis] explode all trees <br> #3 #1 OR #2 <br> #4 MeSH descriptor: [Femur Head] explode all trees <br> #5 #3 AND #4 <br> #6 MeSH descriptor: [Femur Head Necrosis] explode all trees <br> #7 (((femur OR femoral) NEXT head)):ti,ab,kw <br> #8 (((avascular OR aseptic OR ischemic) NEXT (necrosis OR necroses))):ti,ab,kw <br> #9 (osteonecrosis OR osteonecroses OR osteoradionecrosis OR osteoradionecroses):ti,ab,kw <br> #10 #8 OR #9 <br> #11 #7 AND #10 |

| | |
|---|---|
| #12 | ("ONFH"):ti,ab,kw |
| #13 | #5 OR #6 OR #11 OR #12 |
| #14 | MeSH descriptor: [Diagnostic Imaging] explode all trees |
| #15 | (((diagnostic OR "magnetic resonance" OR medical) NEXT imag*)):ti,ab,kw |
| #16 | (mri* OR tomograph* OR radiograph* OR ultrasonograph* OR x-ray):ti,ab,kw |
| #17 | (((CT OR CAT) NEXT scan*)):ti,ab,kw |
| #18 | #14 OR #15 OR #16 OR #17 |
| #19 | MeSH descriptor: [Sensitivity and Specificity] explode all trees |
| #20 | (sensitivity OR specificity):ti,ab,kw |
| #21 | (pretest NEXT probability):ti,ab,kw |
| #22 | ("post-test" NEXT probability):ti,ab,kw |
| #23 | (predictive NEXT value*):ti,ab,kw |
| #24 | (likelihood NEXT ratio*):ti,ab,kw |
| #25 | ("diagnostic accuracy"):ti,ab,kw |
| #26 | #19 OR #20 OR #21 OR #22 OR #23 OR #24 OR #25 |
| #27 | #13 AND #18 AND #26 |

*Key Question 2: Optimal management of ARCO Stage I-II*

| Ovid MEDLINE(R) ALL | |
|---|---|
| 1 | osteonecrosis/ |
| 2 | exp Osteoradionecrosis/ |
| 3 | 1 or 2 |

| | |
|---|---|
| 4 | exp Femur Head/ |
| 5 | 3 and 4 |
| 6 | exp Femur Head Necrosis/ |
| 7 | ((femur or femoral) adj2 head).tw,kw. |
| 8 | ((avascular or aseptic or ischemic) adj2 (necrosis or necroses)).tw,kw. |
| 9 | (osteonecrosis or osteonecroses or osteoradionecrosis or osteoradionecroses).tw,kw. |
| 10 | 8 or 9 |
| 11 | 7 and 10 |
| 12 | onfh.tw,kw. |
| 13 | 5 or 6 or 11 or 12 |
| 14 | ((fracture* or collapse*) adj2 (femur or femoral) adj2 head*).tw,kw. |
| 15 | exp Femur Head/ |
| 16 | exp Hip Fractures/ |
| 17 | 15 and 16 |
| 18 | 14 or 17 |
| 19 | without.mp. |
| 20 | 18 and 19 |
| 21 | (ARCO adj5 ("1" or "2" or One or Two)).tw,kw. |
| 22 | ("pre-collapse" or precollapse or (before adj2 collaps*)).tw,kw. |
| 23 | 20 or 21 or 22 |
| 24 | 13 and 23 |
| 25 | exp Animals/ not exp Humans/ |
| 26 | 24 not 25 |
| 27 | exp Adult/ |
| 28 | exp Child/ or exp Infant/ or exp Adolescent/ |
| 29 | 28 not 27 |

| | |
|---|---|
| 30 | 26 not 29 |
| 31 | "case report".ti. |
| 32 | (editorial or letter or commentary or note).pt. |
| 33 | 31 or 32 |
| 34 | 30 not 33 |

PubMed

((((((("Osteonecrosis"[Mesh:NoExp]) OR "Osteoradionecrosis"[Mesh]) AND ("Femur Head"[Mesh])) OR ("Femur Head Necrosis"[Mesh])) OR (("femur head"[tw] OR "femoral head"[tw]) AND (("avascular necrosis"[tw] OR "avascular necrosis"[tw] OR "ischemic necrosis"[tw] OR "avascular necroses"[tw] OR "avascular necroses"[tw] OR "ischemic necroses"[tw]) OR (osteonecrosis[tw] OR osteonecroses[tw] OR osteoradionecrosis[tw] OR osteoradionecroses[tw])))) OR (onfh[tw])) AND ((("femur head fracture"[tw] OR "femoral head fracture"[tw] OR "femur head fracture"[tw] OR "femoral head collapse"[tw]) OR (("Femur Head"[Mesh]) AND "Hip Fractures"[Mesh]) AND "without"[tiab]) OR "ARCO 1"[tw] OR "ARCO 2"[tw] OR "ARCO One"[tw] OR "ARCO Two"[tw] "pre-collapse"[tiab] OR "precollapse"[tiab] OR "before collapse"[Title/Abstract:~2]) NOT ("Animals"[MeSH] NOT "Humans"[MeSH])

Embase Classic+Embase (via Ovid)

| | |
|---|---|
| 1 | bone necrosis/ |
| 2 | exp osteoradionecrosis/ |
| 3 | 1 or 2 |
| 4 | exp femoral head/ |
| 5 | 3 and 4 |
| 6 | exp femur head necrosis/ |

| | |
|---|---|
| 7 | ((femur or femoral) adj2 head).tw,kw. |
| 8 | ((avascular or aseptic or ischemic) adj2 (necrosis or necroses)).tw,kw. |
| 9 | (osteonecrosis or osteonecroses or osteoradionecrosis or osteoradionecroses).tw,kw. |
| 10 | 8 or 9 |
| 11 | 7 and 10 |
| 12 | onfh.tw,kw. |
| 13 | 5 or 6 or 11 or 12 |
| 14 | ((fracture* or collapse*) adj2 (femur or femoral) adj2 head*).tw,kw. |
| 15 | exp femoral head/ |
| 16 | exp hip fracture/ |
| 17 | 15 and 16 |
| 18 | 14 or 17 |
| 19 | without.mp. |
| 20 | 18 and 19 |
| 21 | (ARCO adj5 ("1" or "2" or One or Two)).tw,kw. |
| 22 | ("pre-collapse" or precollapse or (before adj2 collaps*)).tw,kw. |
| 23 | 20 or 21 or 22 |
| 24 | 13 and 23 |
| 25 | exp animal/ not exp human/ |
| 26 | 24 not 25 |
| 27 | exp adult/ |
| 28 | exp child/ or exp infant/ or exp adolescent/ |
| 29 | 28 not 27 |
| 30 | 26 not 29 |
| 31 | (letter or commentary or editorial).pt. |
| 32 | 30 not 31 |

| |
|---|
| Web of Science (Core Collection: SCI-EXPANDED, SSCI, AHCI, CPCI-S, CPCI-SSH, BKCI-SSH, ESCI, CCR-EXPANDED, IC) |
| TS=(((("femur head" OR "femoral head") NEAR/3 necrosis) OR osteoradionecrosis OR osteoradionecroses OR ONFH) AND ((("femur head" OR "femur collapse" OR "femoral collapse") AND without) OR (ARCO NEAR/5(1 OR 2 OR One OR Two)) OR "pre-collapse" OR "before collapse" )) |
| Scopus |
| ((((INDEXTERMS("femur head necrosis")) OR (INDEXTERMS("Osteoradionecrosis" OR "Bone Necrosis" OR "Osteonecrosis") AND INDEXTERMS("Femur Head" OR "Femoral Head")) OR ((TITLE-ABS-KEY(((femur OR femoral) W/2 head))) AND (TITLE-ABS-KEY(((avascular OR aseptic OR ischemic) W/2 (necrosis OR necroses)) OR osteonecrosis OR osteonecroses OR osteoradionecrosis OR osteoradionecroses)) OR TITLE-ABS (ONFH))) AND ((((INDEXTERMS("femur head" AND ("hip fractures" OR "hip fracture"))) OR (TITLE-ABS-KEY(((fracture OR collapse) W/2(femur OR femoral)) w/2 head ))) AND (TITLE-ABS(without))) OR (TITLE-ABS-KEY(ARCO w/5 (1 OR 2 OR one OR two))) OR (TITLE-ABS-KEY("pre-collapse" OR precollapse)))) AND NOT (INDEXTERMS(animal OR animals) AND NOT INDEXTERMS(human OR humans))) AND NOT ((INDEXTERMS(Child OR infant OR adolescent)) AND NOT (INDEXTERMS(adult))) |
| Global Index Medicus |
| ((((mh:(Osteoradionecrosis)) OR mh:(Osteonecrosis)) AND (mh:(Femur Head))) OR (mh:(Femur Head Necrosis)) OR ((tw:(osteonecrosis OR osteonecroses OR osteoradionecrosis OR osteoradionecroses OR necrosis OR necroses)) AND (tw:(femur OR femoral))) OR (tw:(onfh))) AND ((tw:("ARCO 1" OR "ARCO One" OR "ARCO 2" OR "ARCO Two")) OR (((tw:(fracture* AND (femur OR femoral) AND head)) OR ((mh:(Femur Head)) AND |

| |
|---|
| (mh:(Hip Fractures)))) AND (tw:(without))) OR (tw:("pre-collapse" OR precollapse OR "before collapse"))) AND NOT ((mh:(Adult)) AND NOT ((mh:(Child)) OR (mh:(Infant)) OR (mh:(Adolescent)))) AND NOT ((mh:(Animals)) AND NOT (mh:(Humans))) |
| Cochrane Library (via Wiley) |
| #1 MeSH descriptor: [Osteonecrosis] this term only<br>#2 MeSH descriptor: [Osteoradionecrosis] explode all trees<br>#3 #1 OR #2<br>#4 MeSH descriptor: [Femur Head] explode all trees<br>#5 #3 AND #4<br>#6 MeSH descriptor: [Femur Head Necrosis] explode all trees<br>#7 (((femur OR femoral) NEXT head)):ti,ab,kw<br>#8 (((avascular OR aseptic OR ischemic) NEXT (necrosis OR necroses))):ti,ab,kw<br>#9 (osteonecrosis OR osteonecroses OR osteoradionecrosis OR osteoradionecroses):ti,ab,kw<br>#10 #8 OR #9<br>#11 #7 AND #10<br>#12 ("ONFH"):ti,ab,kw<br>#13 #5 OR #6 OR #11 OR #12<br>#14 (((fracture* OR collapse*) NEXT (femur headOR femoral head))):ti,ab,kw<br>#15 MeSH descriptor: [Femur Head] explode all trees<br>#16 MeSH descriptor: [Hip Fractures] explode all trees<br>#17 #15 AND #16<br>#18 #14 OR #17<br>#19 (without):ti,ab,kw |

| | |
|---|---|
| #20 | #18 AND #19 |
| #21 | (ARCO 1 OR ARCO 2 OR ARCO One OR ARCO Two):ti,ab,kw |
| #22 | ("pre-collapse" OR precollapse OR (before NEXT collaps*)):ti,ab,kw |
| #23 | #20 OR #21 OR #22 |
| #24 | #13 AND #23 |
| #25 | MeSH descriptor: [Adult] explode all trees |
| #26 | MeSH descriptor: [Child] explode all trees |
| #27 | MeSH descriptor: [Infant] explode all trees |
| #28 | MeSH descriptor: [Adolescent] explode all trees |
| #29 | #26 OR #27 OR #28 |
| #30 | #29 NOT #25 |
| #31 | #24 NOT #30 |

*Key Question 3: Surgical Treatment of ARCO stage III*

| Ovid MEDLINE(R) ALL | |
|---|---|
| 1 | osteonecrosis/ |
| 2 | exp Osteoradionecrosis/ |
| 3 | 1 or 2 |
| 4 | exp Femur Head/ |
| 5 | 3 and 4 |
| 6 | exp Femur Head Necrosis/ |
| 7 | ((femur or femoral) adj2 head).tw,kw. |
| 8 | ((avascular or aseptic or ischemic) adj2 (necrosis or necroses)).tw,kw. |

| | |
|---|---|
| 9 | (osteonecrosis or osteonecroses or osteoradionecrosis or osteoradionecroses).tw,kw. |
| 10 | 8 or 9 |
| 11 | 7 and 10 |
| 12 | onfh.tw,kw. |
| 13 | 5 or 6 or 11 or 12 |
| 14 | (ARCO adj5 ("3" or three or "4" or four)).tw,kw. |
| 15 | (fracture* adj2 (femur or femoral) adj2 head*).tw,kw. |
| 16 | exp Femur Head/ |
| 17 | exp Hip Fractures/ |
| 18 | 16 and 17 |
| 19 | 14 or 15 or 18 |
| 20 | exp Orthopedics/ |
| 21 | exp Orthopedic Procedures/ |
| 22 | (osteotom* or hemiarthroplast* or arthroplast*).tw,kw. |
| 23 | (surger* or surgical).tw,kw. |
| 24 | surgery.fs. |
| 25 | 20 or 21 or 22 or 23 or 24 |
| 26 | 13 and 19 and 25 |
| 27 | exp animals/ not exp humans/ |
| 28 | 26 not 27 |
| 29 | exp adult/ |
| 30 | exp child/ or exp infant/ or exp adolescent/ |
| 31 | 30 not 29 |
| 32 | 28 not 31 |
| 33 | "case report".ti. |
| 34 | (editorial or commentary or comment or letter or review).pt. |

| | |
|---|---|
| 35 | 33 or 34 |
| 36 | 32 not 35 |

PubMed

((((((((("Osteonecrosis"[Mesh:NoExp]) OR "Osteoradionecrosis"[Mesh]) AND ("Femur Head"[Mesh])) OR ("Femur Head Necrosis"[Mesh])) OR (("femur head"[tiab] OR "femoral head"[tiab]) AND (("avascular necrosis"[tiab] OR "avascular necrosis"[tiab] OR "ischemic necrosis"[tiab] OR "avascular necroses"[tiab] OR "avascular necroses"[tiab] OR "ischemic necroses"[tiab]) OR (osteonecrosis[tiab] OR osteonecroses[tiab] OR osteoradionecrosis[tiab] OR osteoradionecroses[tiab])))) OR (onfh[tiab])) AND ((("femur head fracture"[tiab] OR "femoral head fracture"[tiab] OR "femur head collapse"[tiab] OR "femoral head collapse"[tiab]) OR (("Femur Head"[Mesh]) AND "Hip Fractures"[Mesh])) OR ((ARCO[tiab] AND 3[tiab]) OR(ARCO[tiab] AND 4[tiab])))) AND (((("Orthopedics"[Mesh] OR "Orthopedic Procedures"[Mesh]) OR (osteotom*[tiab] OR hemiarthroplast*[tiab] OR arthroplast*[tiab])) OR (surger*[tiab] OR surgical[tiab])) OR ("surgery" [Subheading]))) NOT ("animals"[Mesh] NOT "humans"[Mesh])) NOT (("child"[Mesh] OR "infant"[Mesh] OR "adolescent"[Mesh]) NOT ("adult"[MeSH]))) NOT (editorial[pt] OR letter[pt])

Embase Classic+Embase (via Ovid)

| | |
|---|---|
| 1 | bone necrosis/ |
| 2 | exp osteoradionecrosis/ |
| 3 | 1 or 2 |
| 4 | exp femoral head/ |
| 5 | 3 and 4 |
| 6 | exp femur head necrosis/ |
| 7 | ((femur or femoral) adj2 head).tw,kw. |

| | |
|---|---|
| 8 | ((avascular or aseptic or ischemic) adj2 (necrosis or necroses)).tw,kw. |
| 9 | (osteonecrosis or osteonecroses or osteoradionecrosis or osteoradionecroses).tw,kw. |
| 10 | 8 or 9 |
| 11 | 7 and 10 |
| 12 | onfh.tw,kw. |
| 13 | 5 or 6 or 11 or 12 |
| 14 | (ARCO adj5 ("3" or three or "4" or four)).tw,kw. |
| 15 | (fracture* adj2 (femur or femoral) adj2 head*).tw,kw. |
| 16 | exp femoral head/ |
| 17 | exp hip fracture/ |
| 18 | 16 and 17 |
| 19 | 14 or 15 or 18 |
| 20 | exp orthopedics/ |
| 21 | exp orthopedic surgery/ |
| 22 | (osteotom* or hemiarthroplast* or arthroplast*).tw,kw. |
| 23 | (surger* or surgical).tw,kw. |
| 24 | surgery.fs. |
| 25 | 20 or 21 or 22 or 23 or 24 |
| 26 | 13 and 19 and 25 |
| 27 | exp animal/ not exp human/ |
| 28 | 26 not 27 |
| 29 | exp adult/ |
| 30 | exp child/ or exp infant/ or exp adolescent/ |
| 31 | 30 not 29 |
| 32 | 28 not 31 |
| 33 | (letter or commentary or editorial).pt. |

| |
|---|
| 34 32 not 33 |
| Web of Science (Core Collection: SCI-EXPANDED, SSCI, AHCI, CPCI-S, CPCI-SSH, BKCI-SSH, ESCI, CCR-EXPANDED, IC) |
| TS=(((("femur head" OR "femoral head") NEAR/3 (necrosis OR necroses) OR osteoradionecrosis OR osteoradionecroses OR osteonecrosis OR osteonecroses) OR (ONFH)) AND ((femur head fracture) OR (ARCO NEAR/5 (3 OR three OR 4 OR four))) AND (orthopedic* OR surgery OR surgical OR "orthopedic procedure" OR osteotom* OR hemiarthroplast* OR arthroplast*)) |
| Scopus |
| ((((INDEXTERMS("femur head necrosis")) OR (INDEXTERMS("Osteoradionecrosis" OR "Bone Necrosis" OR "Osteonecrosis") AND INDEXTERMS("Femur Head" OR "Femoral Head")) OR ((TITLE-ABS-KEY(((femur OR femoral) W/2 head))) AND (TITLE-ABS-KEY(((avascular OR aseptic OR ischemic) W/2 (necrosis OR necroses)) OR osteonecrosis OR osteonecroses OR osteoradionecrosis OR osteoradionecroses)) OR TITLE-ABS (ONFH))) AND ((TITLE-ABS-KEY(ARCO w/5 (3 OR three OR 4 OR four))) OR (TITLE-ABS-KEY(((fracture OR collapse) W/2(femur OR femoral)) w/2 head)) OR (INDEXTERMS("femoral head" AND ("hip fracture" OR "hip fractures")))) AND ((INDEXTERMS(orthopedics OR "orthopedic procedures" OR "orthopedic surgery")) OR (TITLE-ABS-KEY(osteotom* OR hemiarthroplast* OR arthroplast OR surgery OR surgical)))) AND NOT ((INDEXTERMS(animal OR animals)) AND NOT (INDEXTERMS(human OR humans)))) AND NOT ((INDEXTERMS(Child OR infant OR adolescent)) AND NOT (INDEXTERMS(adult))) |
| Global Index Medicus |

| |
|---|
| (((mh:(Osteoradionecrosis)) AND (mh:(Femur Head))) OR (mh:(Femur Head Necrosis)) OR ((tw:(osteonecrosis OR osteonecroses OR osteoradionecrosis OR osteoradionecroses OR necrosis OR necroses)) AND (tw:(femur OR femoral))) OR (tw:(onfh))) AND ((tw:("ARCO 3" OR "ARCO Three" OR "ARCO 4" OR "ARCO Four")) OR (tw:(fracture* AND (femur OR femoral) AND head)) OR ((mh:(Femur Head)) AND (mh:(Hip Fractures)))) AND ((mh:(Orthopedics)) OR (mh:(Orthopedic Procedures)) OR (mh:(Surgery)) OR (tw:(osteotom* OR hemiarthroplast* OR arthroplast* OR surger* OR surgical))) AND NOT ((mh:(Adult)) AND NOT ((mh:(Child)) OR (mh:(Infant)) OR (mh:(Adolescent)))) AND NOT ((mh:(Animals)) AND NOT (mh:(Humans))) |
| Cochrane Library (via Wiley) |
| #1 MeSH descriptor: [Osteonecrosis] this term only<br>#2 MeSH descriptor: [Osteoradionecrosis] explode all trees<br>#3 #1 OR #2<br>#4 MeSH descriptor: [Femur Head] explode all trees<br>#5 #3 AND #4<br>#6 MeSH descriptor: [Femur Head Necrosis] explode all trees<br>#7 (((femur OR femoral) NEXT head)):ti,ab,kw<br>#8 (((avascular OR aseptic OR ischemic) NEXT (necrosis OR necroses))):ti,ab,kw<br>#9 (osteonecrosis OR osteonecroses OR osteoradionecrosis OR osteoradionecroses):ti,ab,kw<br>#10 #8 OR #9<br>#11 #7 AND #10<br>#12 ("ONFH"):ti,ab,kw<br>#13 #5 OR #6 OR #11 OR #12<br>#14 (ARCO 3 OR ARCO three OR ARCO 4 OR ARCO four):ti,ab,kw<br>#15 ((((femur OR femoral) NEXT head))):ti,ab,kw |

| | |
|---|---|
| #16 | MeSH descriptor: [Femur Head] explode all trees |
| #17 | MeSH descriptor: [Hip Fractures] explode all trees |
| #18 | #16 AND #17 |
| #19 | #14 OR #15 OR #18 |
| #20 | MeSH descriptor: [Orthopedics] explode all trees |
| #21 | MeSH descriptor: [Orthopedic Procedures] explode all trees |
| #22 | (osteotom* OR hemiarthroplast* OR arthroplast*):ti,ab,kw |
| #23 | (surger* OR surgical):ti,ab,kw |
| #24 | MeSH descriptor: [] explode all trees and with qualifier(s): [surgery - SU] |
| #25 | #20 OR #21 OR #22 OR #23 OR #24 |
| #26 | #13 AND #19 AND #25 |
| #27 | MeSH descriptor: [Adult] explode all trees |
| #28 | MeSH descriptor: [Child] explode all trees |
| #29 | MeSH descriptor: [Infant] explode all trees |
| #30 | MeSH descriptor: [Adolescent] explode all trees |
| #31 | #28 OR #29 OR #30 |

Supplemental Table S1. Study Selection Criteria for Systematic Review: Diagnosis and Treatment of Osteonecrosis of Femoral Head

| **Key Question Number** | **Inclusion Criteria** | **Exclusion Criteria** |
|---|---|---|
| **KQ #1** | • Focus of article is on imaging, not on the treatment outcome<br>• Humans studies only<br>• Adult population only (age >16 years) | • Foreign language without full text English translation<br>• Single case reports<br>• Interventions evaluated in a single, single-center study with <100 patients |

| | | |
|---|---|---|
| | | • Articles that do not present original data (e.g., reviews, editorials) |
| **KQ #2** | • ARCO Stages I-II (pre-collapse) ONFH<br>• Evidence level 1–4<br>• Case series >15 patients<br>• Human only<br>• Prospective or retrospective<br>• Non-traumatic ONFH<br>• Age >16 year<br>• Advanced imaging (CT or MRI) in addition to XR, used to assess for subchondral collapse<br>• Treatment intervention is uniform across entire treatment cohort and evaluated in at least 2 independent publications for the same outcome.<br>• Outcome includes radiographic progression, not only conversion to total hip arthroplasty<br>• Follow up: >2 years | • Animal studies<br>• Foreign language without full text English translation<br>• Single case reports<br>• No duplicate papers or patient cohorts<br>• Articles that do not present original data (e.g., reviews, editorials) |
| **KQ #3** | • ARCO Stage III ONFH<br>• Evidence level 1–4<br>• Case series >15 patients<br>• Human only<br>• Surgical interventions only<br>• Prospective or retrospective<br>• Non-traumatic ONFH<br>• Age >16 years<br>• Staging data provided<br>• Treatment intervention is uniform across entire treatment cohort<br>• Outcome data provided<br>• Follow up: >2 years | • Foreign language without full text English translation<br>• Single case reports<br>• No duplicate papers or patient cohorts<br>• Articles that do not present original data (e.g., reviews, editorials) |

ARCO- Association Research Circulation Osseous; CT- computed tomography; MRI- magnetic resonance imaging; ONFH- osteonecrosis of femoral head

Supplemental Figure S1 PRISMA Flow Diagram for Study Selection in Primary Searches

(A) Key Question 1: Diagnostic Imaging

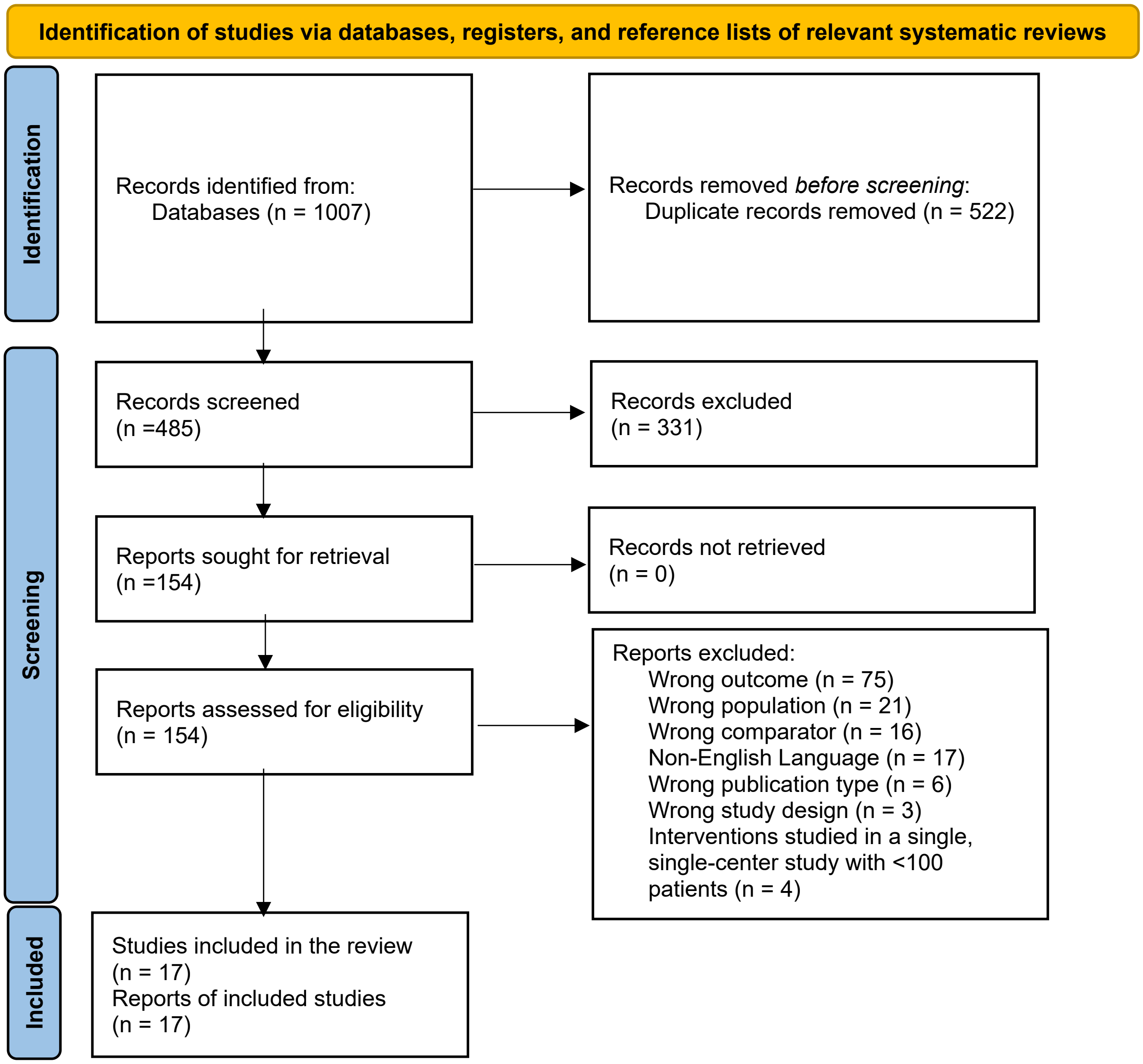

(B) Key Question 2: Optimal Management of ARCO Stage I-II Osteonecrosis of Femoral Head

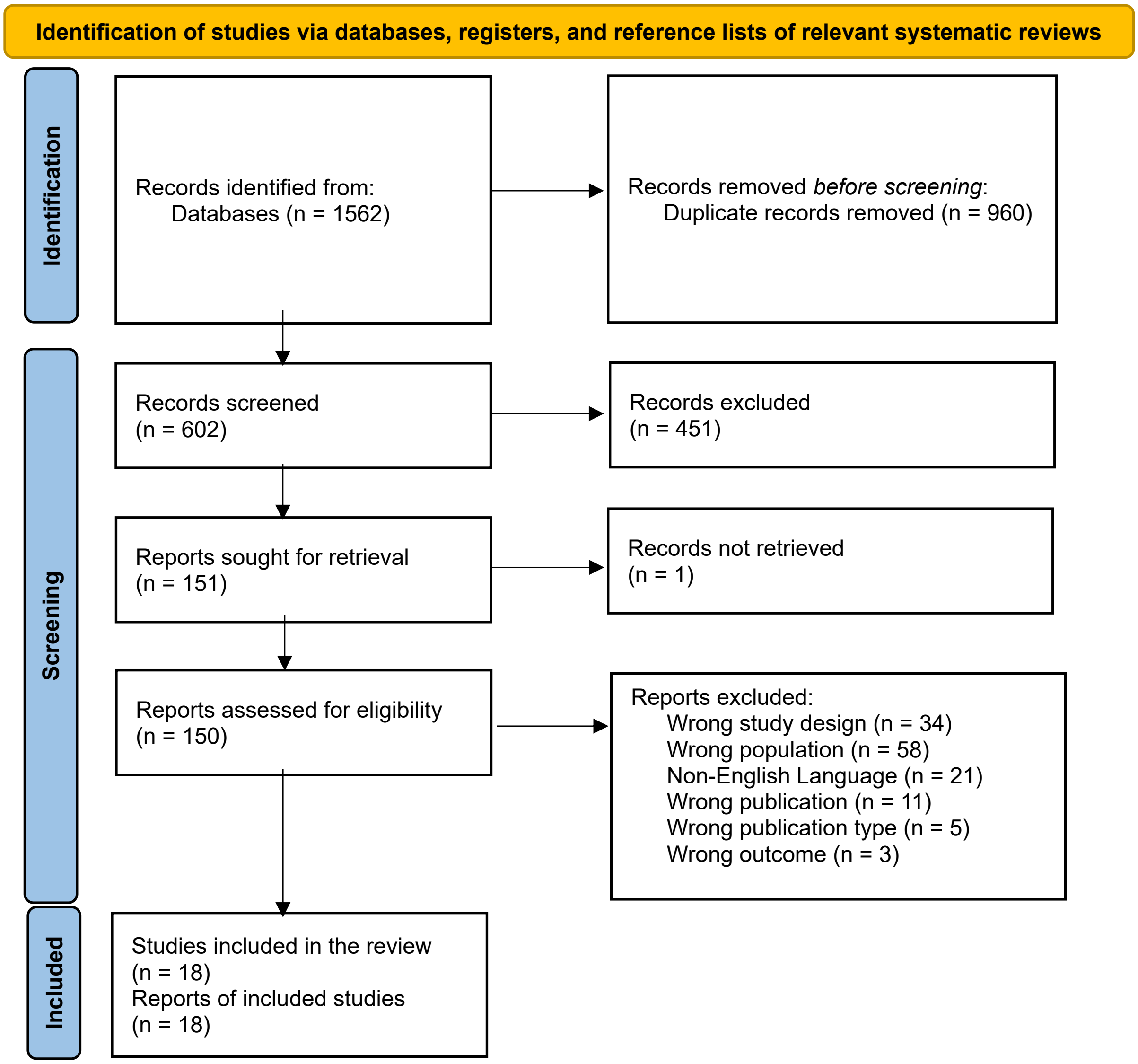

(C) Key Question 3: Surgical Treatment of ARCO Stage III Osteonecrosis of Femoral Head

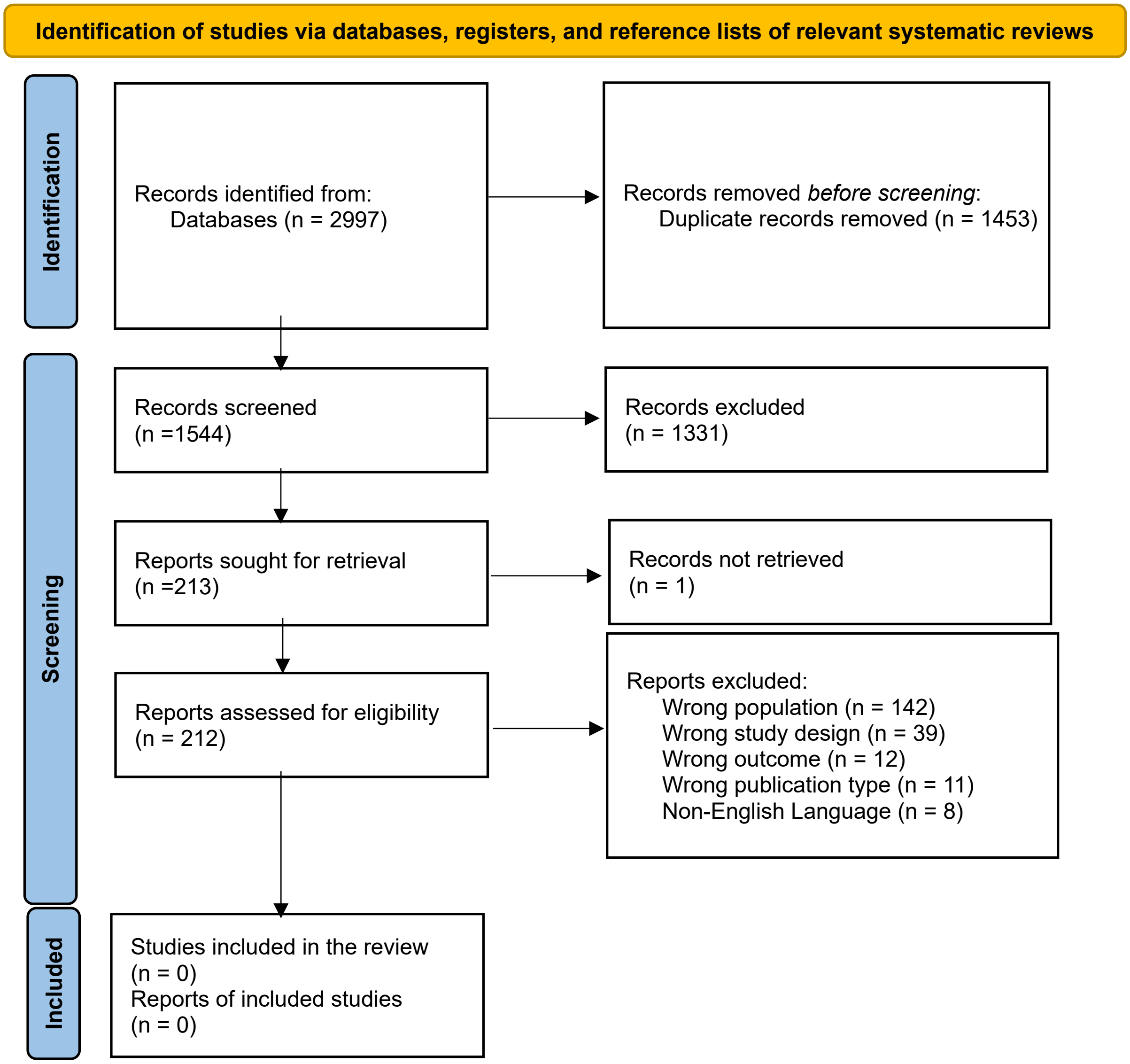

**S2. Description of Population, Intervention, Comparator, and Outcomes in Included Studies**

Supplemental Table S2 Characteristic features of studies that were used in PICO1 recommendations

| **Pico 1a:** Optimal imaging modality for diagnosis of ONFH | | | | | | |
|---|---|---|---|---|---|---|
| **Authors, Study (PMID)** | **Country (year)** | **Sample size** | **Study design** | **Reference standard** | **Modalities** | **Main outcomes** |
| **Ryu et al.**, Bone SPECT is more sensitive than MRI in the detection of early osteonecrosis of the femoral head after renal transplantation (12163624) | Korea (2002) | 48 | Retrospective comparative | Histology | SPECT vs. MRI | Sensitivity, TP*, FP*, TN*, FN* |
| **Miller et al.**, Femoral Head Osteonecrosis<br>Detection by Magnetic Resonance Imaging Versus Single-Photon Emission Computed Tomography (2791384) | USA (1989) | 56 | Retrospective comparative | Histology | SPECT vs. MRI | Sensitivity, Specificity, TP*, FP*, TN*, FN* |
| **Stulberg et al.**, Multimodality Approach to Osteonecrosis of the Femoral Head (2783898) | USA (1989) | 80 | Retrospective comparative | Histology | SPECT vs. MRI | Sensitivity, Specificity,<br>TP*, FP*, TN*, FN* |
| **Beltran et al.,** Femoral Head Avascular Necrosis: MR Imaging with Clinical Pathologic and Radionuclide Correlation (3336682) | USA (1988) | 85 | Retrospective single-arm | Histology | BS | Sensitivity, Specificity, TP*, FP*, TN*, FN* |
| **Thickman et al.,** Magnetic resonance imaging of | USA (1986) | 90 | Retrospective comparative | Histology | MRI vs. BS | Sensitivity, Specificity, |

| | | | | | | |
|---|---|---|---|---|---|---|
| avascular necrosis of the femoral head (3961519) | | | | | | TP*, FP*, TN*, FN* |
| **Bassett et al.,** Magnetic resonance imaging in the early diagnosis of ischemic necrosis of the femoral head. Preliminary results (3791749) | USA (1987) | 50 | Retrospective comparative | - | MRI vs. BS and X-ray | Sensitivity*, Specificity*, TP*, FP*, TN*, FN* |
| **Ge et al.,** X-ray, digital tomographic fusion, CT, and MRI in early ischemic necrosis of the femoral head (38215113) | China (2024) | 440 | Retrospective comparative | - | MRI vs. CT, DTS, and X-ray | Sensitivity, Specificity, TP*, FP*, TN*, FN* |
| **Agarwal et al.,** Incremental value of 99mTc-MDP hybrid SPECT/CT over planar scintigraphy and SPECT in avascular necrosis of the femoral head (26308940) | India (2015) | 64 | Retrospective comparative | MRI or Histology | SPECT/CT vs. SPECT and BS | Sensitivity, Specificity, TP*, FP*, TN*, FN* |
| **Collier et al.,** Detection of Femoral Head Avascular Necrosis in Adults by SPECT (3875700) | USA (1985) | 20 | Retrospective comparative | Histology | SPECT vs. X-ray and BS | Sensitivity* |
| **PICO 1b:** Optimal imaging modality for detecting subchondral fracture in ONFH | | | | | | |
| **Jordan et al.,** Femoral Head Osteonecrosis: Computed Tomography Not Needed to Identify Collapse When Using the Association Research Circulation Osseous Staging System (37867923) | USA (2023) | 228 | Retrospective comparative | CT, MRI, or X-ray | CT vs 3-T MRI | Diagnostic yield for ARCO stage III ONFH |
| **Yang et al.,** The Value of the Frog Lateral View Radiograph for Detecting | China (2022) | 1001 | Retrospective comparative | CT or MRI | Frog view X-ray vs. AP X-ray | Sensitivity, Specificity, |

| | | | | | | |
|---|---|---|---|---|---|---|
| Collapse of Femur Head Necrosis: A Retrospective Study of 1001 Cases (35425771) | | | | | | NPV, PPV, Accuracy |
| **Chee et al.,** Diagnostic accuracy of digital radiography for the diagnosis of osteonecrosis of the femoral head, revisited (30326729) | Korea (2019) | 402 | Retrospective comparative | MRI | Digital X-ray vs. Frog view X-ray | Sensitivity, Specificity |
| **Stevens et al,** Subchondral fractures in osteonecrosis of the femoral head: comparison of radiography, CT, and MR imaging (12540435) | USA (2003) | 45 | Retrospective comparative | CT | X-ray vs. 1.5-T MRI | Sensitivity, Specificity, NPV, PPV, |
| **PICO 1c:** Optimal imaging modality for monitoring the effect of any intervention for treating ONFH | | | | | | |
| **Thickman et al.,** Magnetic resonance imaging of avascular necrosis of the femoral head (3961519) | USA (1986) | 90 | Retrospective comparative | Histology | MRI vs. BS | Necrosis volume |
| **Plenk et al.,** Magnetic Resonance Imaging and Histology of Repair in Femoral Head Osteonecrosis (11347847) | Austria (2001) | 13 | Retrospective single-arm | Histology | MRI | Necrosis volume |
| **Bassounas et al.,** Femoral head osteonecrosis: Volumetric MRI assessment and outcome (17555907) | Greece (2007) | 87 | Retrospective single-arm | - | MRI | Necrosis volume |
| **PICO 1d:** Imaging studies to correlate and differentiate symptomatic versus asymptomatic ONFH | | | | | | |

| | | | | | | |
|---|---|---|---|---|---|---|
| **Hatanaka et al.,** Differences in magnetic resonance findings between symptomatic and asymptomatic pre-collapse osteonecrosis of the femoral head (30777197) | Japan (2019) | 123 | Retrospective observational | - | MRI | Correlation with symptoms |
| **Huang et al.,** MR imaging of bone marrow edema and joint effusion in patients with osteonecrosis of the femoral head: relationship to pain (12876044) | China (2003) | 110 | Retrospective observational | - | MRI | Correlation with symptoms |
| **Koo et al.,** Bone marrow edema and associated pain in early-stage osteonecrosis of the femoral head: prospective study with serial MR images (10580944) | Korea (1999) | 37 | Prospective observational | - | MRI | Correlation with symptoms |

SPECT: Single Photon Emission Computed Tomography; MRI: Magnetic Resonance Imaging; BS: Bone Scintigraphy; CT: Computed Tomography; DTS: Digital tomographic fusion; NPV: Negative Predictive Value; PPV: Positive Predictive Value; TP: true positive; FP: false positive; TN: true negative; FN: false negative; * shows the outcomes that were back-calculated.

Supplemental Table S3 Characteristic features of studies that were used in PICO2 recommendations

| **Authors, Study (PMID)** | **Country (year)** | **Main etiology** | **Study design** | **Intervention** No. of hips, Age (y), Follow-up (mo) | **Comparison group** No. of hips, Age (y), Follow-up (mo) | **Main outcomes** |
|---|---|---|---|---|---|---|
| **Gangji et al.,** Autologous bone marrow cell implantation in the treatment of non-traumatic osteonecrosis of the femoral head: Five-year follow-up of a prospective controlled study (21821156) | Belgium (2011) | Corticosteroids | RCT | CD + BMC<br>13<br>42.2 ± 2.6<br>60 | CD<br>11<br>45.7 ± 2.8<br>60 | Progression to collapse |
| **Pepke et al.,** Core Decompression and Autologous Bone Marrow Concentrate for Treatment of Femoral Head Osteonecrosis: A Randomized Prospective Study (27114808) | Germany (2016) | Corticosteroids | RCT | CD + BMC<br>11<br>44.3 ± 3.4<br>24 | CD<br>14<br>44.5 ± 3.3<br>24 | Progression to collapse |
| **Boontanapibul et al.,** Modified Kerboul Angle Predicts Outcome of Core Decompression With or Without Additional Cell Therapy (33618954) | USA (2021) | Corticosteroids | Retrospective comparative cohort | CD + BMC | CD | Progression to collapse |
| **Wan et al.,** Comparison of the outcome of different bone grafts combined with modified core decompression for the treatment of ARCO II stage femoral head necrosis (35536364) | China (2022) | Corticosteroids and alcohol | RCT | VBG<br>46<br>28.83 ± 5.49<br>44.62 ± 1.81 | Non-VBG<br>45<br>29.64 ± 5.43<br>44.36 ± 1.94 | Progression to collapse |
| **Kim et al.,** Vascularized compared with nonvascularized fibular grafts for large osteonecrotic lesions of the femoral head (16140817) | Korea (2005) | Alcohol, corticosteroids, idiopathic | Retrospective comparative cohort | VBG<br>10<br>43 (24-52)<br>50 (36-66) | Non-VBG<br>10<br>44 (23-51)<br>50 (36-67) | Progression to collapse |
| **Plakseychuk et al.,** Vascularized compared with nonvascularized fibular grafting for the treatment of osteonecrosis of the femoral head (12672831) | USA (2003) | Corticosteroids, idiopathic, alcohol | Retrospective comparative cohort | VBG<br>35<br>-<br>60 | Non-VBG<br>35<br>-<br>60 | Progression to collapse |
| **Kawate et al.,** Limitations of Joint-Preserving Treatment for Osteonecrosis of the Femoral Head: Limitation of Free Vascularized Fibular Grafting (No PMID) | Japan (2007) | Corticosteroids and alcohol | Retrospective single-arm study | VBG<br>38<br>39 (22-60)<br>72 (6-144) | - | Progression to collapse |
| **Ozturk et al.,** Clinical results of free vascularized fibula graft in the management of precollapse osteonecrosis of the femoral head: A retrospective clinical study (35416161) | Turkey (2022) | Corticosteroids and alcohol | Retrospective single-arm study | VBG<br>39<br>36 ± 14<br>66 (24-168) | - | Progression to collapse |
| **Soucacos et al.,** Treatment of avascular necrosis of the femoral head with vascularized fibular transplant (11347825) | Greece (2001) | Corticosteroids | Retrospective single-arm study | VBG<br>39<br>32 (16-54)<br>56 (12-120) | - | Progression to collapse |
| **Unal et al.** Treatment of osteonecrosis of the femoral head with free vascularized fibular grafting: Results of 7.6-year follow-up. | Turkey (2016) | Corticosteroids | Retrospective single-arm study | VBG<br>17<br>30.7 (15–53)<br>91 (60-110) | - | Progression to collapse |

| | | | | | | |
|---|---|---|---|---|---|---|
| **Biswal et al.,** Transtrochanteric rotational osteotomy for nontraumatic osteonecrosis of the femoral head in young adults (19142686) | Korea (2009) | Alcohol, corticosteroids, idiopathic | Retrospective single-arm study | TRO<br>36<br>28 (18 – 46)<br>84 (18-156) | - | Progression to collapse |
| **Hamanishi et al.,** The clinical and radiographic results of intertrochanteric curved varus osteotomy for idiopathic osteonecrosis of the femoral head (24394984) | Japan (2014) | Corticosteroids and alcohol | Retrospective single-arm study | ICVO<br>30<br>38 (15–67)<br>75 (12–196 | - | Progression to collapse |
| **Simank et al.,** Comparison of results of core decompression and intertrochanteric osteotomy for nontraumatic osteonecrosis of the femoral head using Cox regression and survivorship analysis (11547379) | Germany (2001) | Corticosteroids | Retrospective comparative cohort | IO<br>29<br>42 (21–60)<br>108 (18–228) | CD<br>32<br>40 (16–77)<br>108 (18–228) | Progression to collapse |
| **Mohanty et al.,** Management of non-traumatic avascular necrosis of the femoral head-a comparative analysis of the outcome of multiple small diameter drilling and core decompression with fibular grafting (27757848) | India (2017) | Idiopathic | Retrospective comparative cohort | CD + Non-VBG<br>28<br>36.67 ± 7.8<br>46 | CD<br>25<br>34.1 ± 7.3<br>76 | Progression to collapse |
| **Hernigou et al.,** The natural history of asymptomatic osteonecrosis of the femoral head in adults with sickle cell disease (17142405) | France (2006) | Sickle cell disease | Prospective observational cohort | No intervention<br>121<br>26 (18-31)<br>168 (120-240) | - | Progression to symptoms |
| **Kang et al.,** The natural history of asymptomatic osteonecrosis of the femoral head (23340674) | Korea (2013) | Alcohol, idiopathic, corticosteroids | Prospective observational cohort | No intervention<br>68<br>49.6 (24 to 82)<br>6.6 (2.0–10.6) | - | Progression to symptoms |
| **Min et al.,** Untreated asymptomatic hips in patients with osteonecrosis of the femoral head (18327630) | Korea (2008) | Alcohol, idiopathic, corticosteroids | Prospective observational cohort | No intervention<br>81<br>50.5 (22–77)<br>100 (60-192) | - | Progression to symptoms |
| **Nam et al.,** Fate of untreated asymptomatic osteonecrosis of the femoral head (18310696) | Korea (2008) | Alcohol, idiopathic, corticosteroids | Prospective observational cohort | No intervention<br>105<br>50 (23-73)<br>94 (10-272) | - | Progression to symptoms |

RCT: Randomized Controlled Trial; CD: Core Decompression; BMC: Bone Marrow Concentrate; VBG: Vascularized Bone Grafting; TRO: Transtrochanteric Rotational Osteotomy; ICVO: Intertrochanteric Curved Varus Osteotomy; IO: Intertrochanteric Osteotomy

## S3. Risk of Bias Assessments

Supplemental Figure S2 Risk of bias assessment using QUADAS-2 tool for studies under Key Question 1

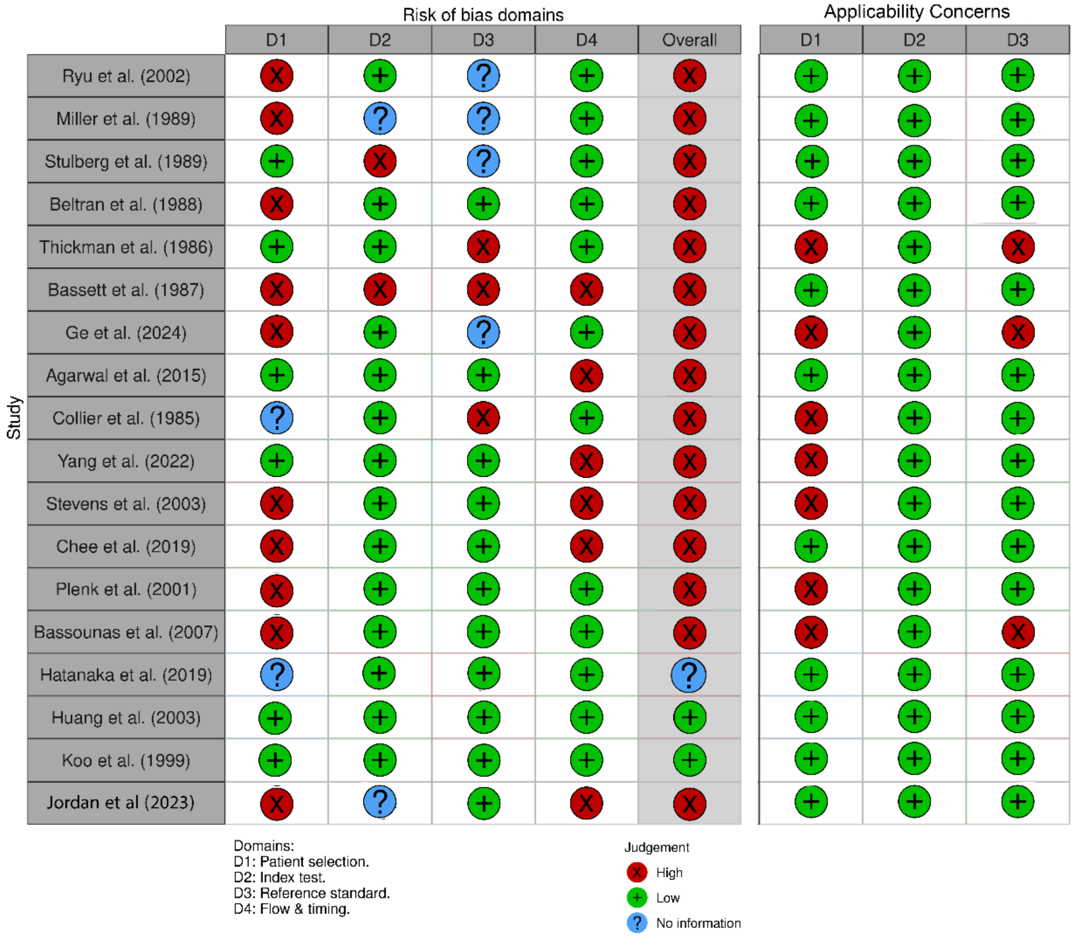


Supplemental Figure S3 Risk of bias assessment using the Cochrane RoB2.0 tool for RCT studies under Key Question 2

Risk of bias domains

| Study | D1 | D2 | D3 | D4 | D5 | Overall |
|---|---|---|---|---|---|---|
| Gangji et al. (2011) | X | - | + | + | + | X |
| Pepke et al. (2016) | - | - | - | - | + | - |
| Wan et al. (2022) | - | + | + | X | - | X |

Domains:
D1: Bias arising from the randomization process.
D2: Bias due to deviations from intended intervention.
D3: Bias due to missing outcome data.
D4: Bias in measurement of the outcome.
D5: Bias in selection of the reported result.

Judgement
X High
- Some concerns
+ Low

Supplemental Figure S4 Risk of bias assessment using the ROBINS-I tool for non-RCT studies under Key Question 2

Risk of bias domains

| Study | D1 | D2 | D3 | D4 | D5 | D6 | D7 | Overall |
|---|---|---|---|---|---|---|---|---|
| Boontanbnapibul et al. (2021) | X | - | + | + | + | - | + | X |
| Kim et al. (2005) | X | - | + | - | + | - | + | X |
| Plakseychuk et al. (2003) | X | - | + | - | + | - | + | X |
| Kawate et al. (2007) | X | - | + | + | + | - | + | X |
| Ozturk et al. (2022) | X | - | + | + | + | - | + | X |
| Soucacos et al. (2001) | X | - | + | + | + | - | + | X |
| Unal et al. (2016) | X | - | + | + | + | - | + | X |
| Biswal et al. (2009) | X | - | + | + | + | - | + | X |
| Hamanishi et al. (2014) | X | - | + | + | + | - | + | X |
| Simank et al. (2001) | X | - | + | - | - | + | + | X |
| Mohanty et al. (2017) | X | - | + | + | + | - | + | X |
| Hernigou et al. (2006) | + | - | + | + | + | - | + | - |
| Min et al. (2008) | + | - | + | + | + | - | + | - |
| Nam et al. (2008) | + | - | + | + | + | - | + | - |
| Kang et al. (2013) | + | - | + | + | + | - | + | - |

Domains:
D1: Bias due to confounding.
D2: Bias due to selection of participants.
D3: Bias in classification of interventions.
D4: Bias due to deviations from intended interventions.
D5: Bias due to missing data.
D6: Bias in measurement of outcomes.
D7: Bias in selection of the reported result.

Judgement
X Serious
- Moderate
+ Low

**S4. Meta-analyses**

Supplemental Figure S5. Summary Receiver Operating Characteristics Plot for Diagnostic Test Accuracy Meta-Analysis for Different Modalities to Diagnose Osteonecrosis of Femoral Head

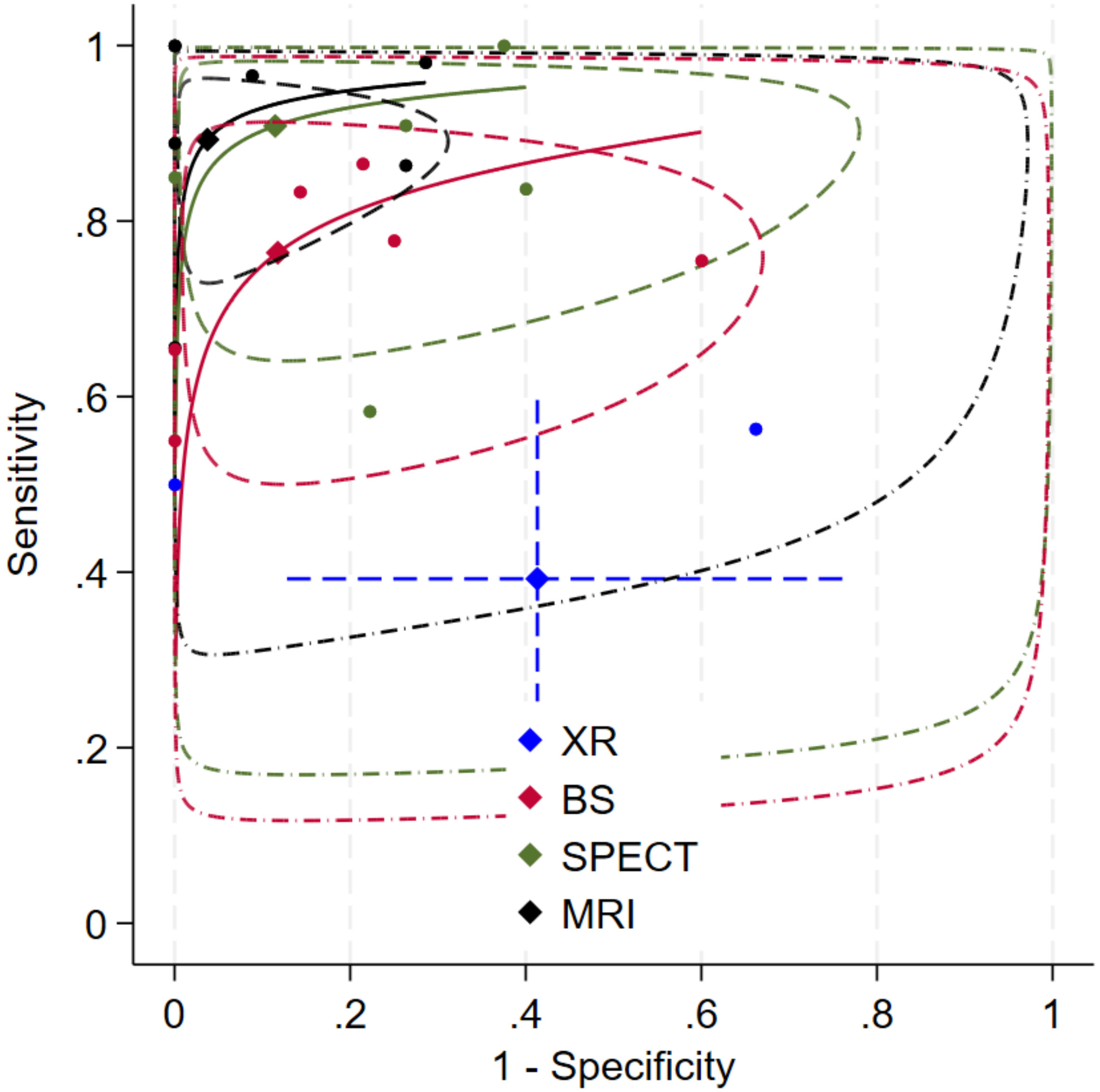


Diamonds- summary estimate

Dashed line (called contours)- 95% CI for the summary estimate

Dash-dot-Dash line (contours)- 95% Prediction interval

As seen in the plot, 95% prediction is very large, indicating “imprecision” in estimate

Supplemental Figure S6. Pooled Specificity and Sensitivity Estimates for KQ1d: Differentiating Asymptomatic versus Symptomatic Nontraumatic ONFH

(A) Bone Marrow Edema

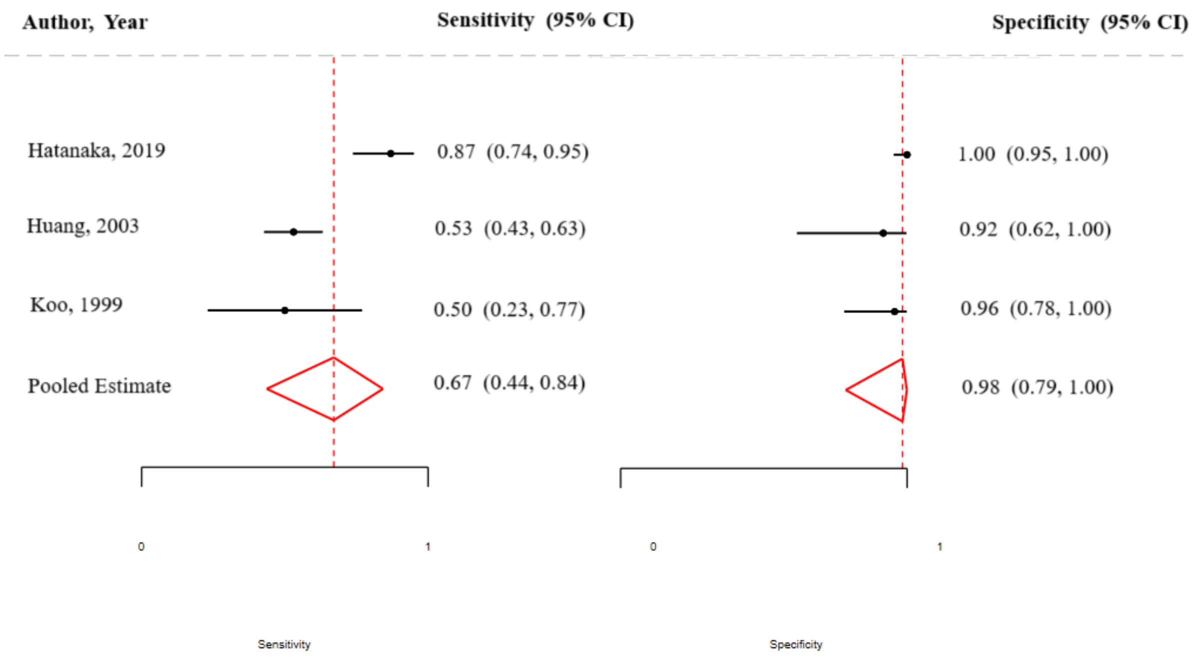


(B) Effusion

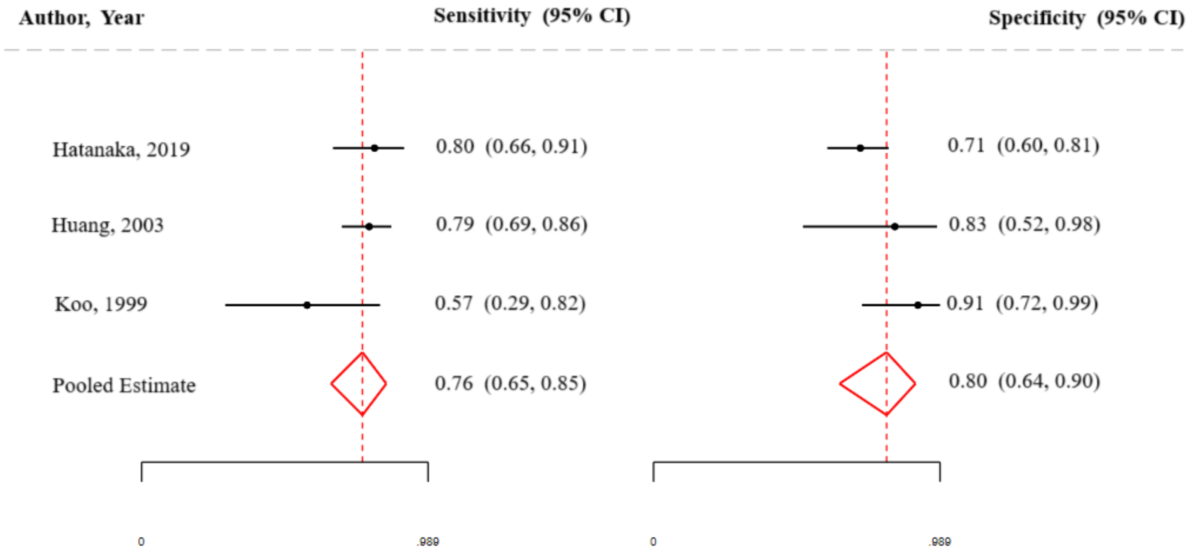

Supplemental Figure S7. Positive Likelihood Ratios for MRI Signs to Differentiate Symptomatic versus Asymptomatic Nontraumatic ONFH

(A). Bone Marrow Edema

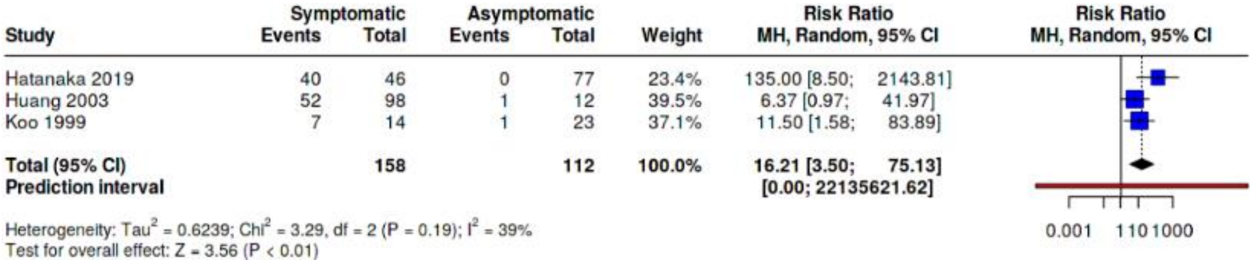


(B). Grade 2+ Joint Effusion

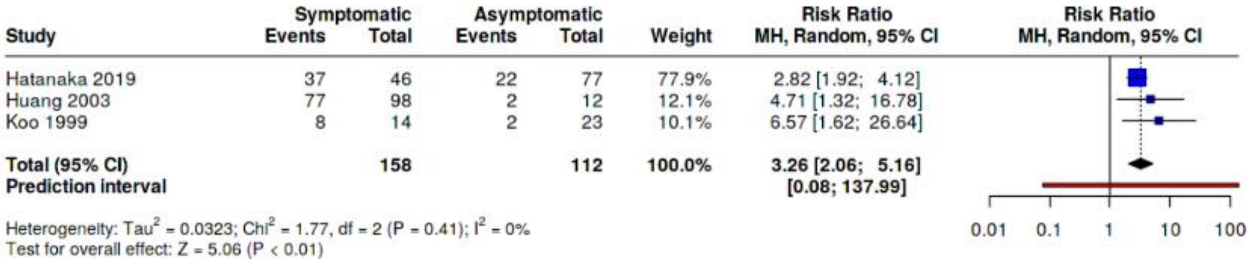


Events = number of hips with positive signs in the group.

Supplemental Table S4. Diagnostic Odds Ratios for MRI signs to Differentiate Between Symptomatic Versus Asymptomatic Nontraumatic ONFH.

| **Study / Pooled Estimate** | **BME DOR** | **BME 95% CI** | **Effusion DOR** | **Effusion 95% CI** |
|---|---|---|---|---|
| Hatanaka | 965.77 | 53.06 – 17,578.19 | 9.74 | 4.10 – 23.11 |
| Huang | 8.66 | 1.51 – 49.65 | 15.14 | 3.52 – 65.17 |
| Koo | 15 | 2.16 – 104.18 | 11.25 | 2.14 – 59.19 |
| Pooled (REML) | 35.68 | 4.50 – 283.08 | 10.97 | 5.57 – 21.63 |
